# Polymeric micelles effectively reprogram the tumor microenvironment to potentiate nano-immunotherapy in mouse breast cancer models

**DOI:** 10.1038/s41467-022-34744-1

**Published:** 2022-11-22

**Authors:** Myrofora Panagi, Fotios Mpekris, Pengwen Chen, Chrysovalantis Voutouri, Yasuhiro Nakagawa, John D. Martin, Tetsuro Hiroi, Hiroko Hashimoto, Philippos Demetriou, Chryso Pierides, Rekha Samuel, Andreas Stylianou, Christina Michael, Shigeto Fukushima, Paraskevi Georgiou, Panagiotis Papageorgis, Petri Ch. Papaphilippou, Laura Koumas, Paul Costeas, Genichiro Ishii, Motohiro Kojima, Kazunori Kataoka, Horacio Cabral, Triantafyllos Stylianopoulos

**Affiliations:** 1 Cancer Biophysics Laboratory, Department of Mechanical and Manufacturing Engineering, University of Cyprus, Nicosia, Cyprus; 2 Department of Bioengineering, Graduate School of Engineering, The University of Tokyo, Bunkyo, Tokyo Japan; 3 Division of Pathology, Exploratory Oncology Research & Clinical Trial Center, National Cancer Center, Kashiwanoha, Kashiwa, Chiba Japan; 4 Department of Integrated Biosciences, Laboratory of Cancer Biology, Graduate School of Frontier Sciences, The University of Tokyo, Kashiwanoha, Kashiwa, Chiba Japan; 5 Division of Innovative Pathology and Laboratory Medicine, Exploratory Oncology Research & Clinical Trial Center, National Cancer Center, Kashiwanoha, Kashiwa, Chiba Japan; 6 The Center for the Study of Hematological and other Malignancies, Nicosia, Cyprus; 7 Center for Stem Cell Research (a unit of inStem Bengaluru), Christian Medical College Campus Bagayam, Vellore, Tamil Nadu India; 8 Basic and Translational Cancer Research Center, School of Sciences, European University of Cyprus, Nicosia, Cyprus; 9 Karaiskakio Foundation, Nicosia, Cyprus; 10 Cyprus Cancer Research Institute, Nicosia, Cyprus; 11 Department of Pathology and Clinical Laboratories, National Cancer Center Hospital East, Kashiwanoha, Kashiwa, Chiba Japan; 12 Innovation Center of NanoMedicine, Kawasaki Institute of Industrial Promotion, Kawasaki, Japan; 13 Institute for Future Initiatives, The University of Tokyo, Bunkyo, Tokyo Japan

**Keywords:** Biopolymers in vivo, Cancer microenvironment, Cancer models, Targeted therapies, Monocytes and macrophages

## Abstract

Nano-immunotherapy improves breast cancer outcomes but not all patients respond and none are cured. To improve efficacy, research focuses on drugs that reprogram cancer-associated fibroblasts (CAFs) to improve therapeutic delivery and immunostimulation. These drugs, however, have a narrow therapeutic window and cause adverse effects. Developing strategies that increase CAF-reprogramming while limiting adverse effects is urgent. Here, taking advantage of the CAF-reprogramming capabilities of tranilast, we developed tranilast-loaded micelles. Strikingly, a 100-fold reduced dose of tranilast-micelles induces superior reprogramming compared to free drug owing to enhanced intratumoral accumulation and cancer-associated fibroblast uptake. Combination of tranilast-micelles and epirubicin-micelles or Doxil with immunotherapy increases T-cell infiltration, resulting in cures and immunological memory in mice bearing immunotherapy-resistant breast cancer. Furthermore, shear wave elastography (SWE) is able to monitor reduced tumor stiffness caused by tranilast-micelles and predict response to nano-immunotherapy. Micellar encapsulation is a promising strategy for TME-reprogramming and SWE is a potential biomarker of response.

## Abstract (web summary)

Nanotherapy has potential utility in cancer, particularly in targeted delivery of therapeutics. Here the authors demonstrate delivery of tranilast loaded micelles to improve the reprogramming of cancer associated fibroblasts and monitor tumour stiffness to predict responses.

## Introduction

Nano-drugs combined with immunotherapy (nano-immunotherapy) have high potential to improve cancer patient outcomes, as already demonstrated in triple-negative breast cancer with the combination of nanoparticle albumin-bound paclitaxel and the immune checkpoint blocker (ICB) antibody, atezolizumab^1–3^. This regimen, however, has a modest effect on survival, with median survival lasting less than two years. Compromised therapeutic outcomes are likely to be caused in part by abnormalities in the tumor microenvironment (TME) that inhibit the delivery of nanoparticles and antibodies and induce hypoxic and immunosuppressive conditions that fuel tumor progression, metastasis and drug resistance^4–6^. TME abnormalities in triple-negative breast cancers include the dense tumor interstitial space, abundant in cancer-associated fibroblasts (CAFs), collagen and hyaluronan, which causes stiffening of the tumor and accumulation of mechanical forces^7–11^. The development of such mechanical forces within the TME leads to tumor vessel compression and thus, the formation of a dysfunctional vasculature that limits tissue oxygenation and drug delivery^12,13^. An established approach aiming to improve the efficacy of drug regimens in such desmoplastic tumor types is the “normalization” of the TME^5^. Normalizing structural components of the tumor stroma results in repairing tumor blood vessel abnormalities, improving perfusion and simultaneously promoting the delivery of nanomedicines in the TME^14–17^. A new and distinct class of molecules possessing TME normalization capabilities are the “mechanotherapeutics”^16^. These agents modulate tumor mechanics to alleviate stiffness and reduce intratumoral mechanical forces in order to decompress tumor vessels and improve perfusion. Mechanotherapeutics act by targeting extracellular matrix components or CAFs^14–16^.

The use of mechanotherapeutics thus far mostly involves the repurposing of already approved drugs. Classes of drugs that have been tested in preclinical studies include anti-fibrotic agents, anti-hypertensives and corticosteroids. From the first class of medications, we successfully repurposed tranilast, an anti-fibrotic and antihistamine drug approved in Japan and South Korea and pirfenidone, a globally approved anti-fibrotic drug for the treatment of idiopathic pulmonary fibrosis^18,19^. These agents were able to reduce stiffness and mechanical forces, improve tumor perfusion and significantly enhance the efficacy of chemotherapy and nanomedicine by affecting CAFs. From the second class of medications, CAFs reprogramming by repurposing the angiotensin receptor blocker, losartan, improved the delivery of chemo- and nano-medicine, causing similar to tranilast and pirfenidone effects^15,20,21^. Bosentan is also another anti-hypertensive that was recently employed to act as a mechanotherapeutic^22^. Whereas the corticosteroid dexamethasone improved the delivery and efficacy of cisplatin nanocarriers^23^. Furthermore, mechanotherapeutics have shown to improve immunostimulation and the efficacy of ICBs in breast cancers, whereas combination of tranilast with nano-immunotherapy has further shown to lead to complete tumor regressions in models of triple negative breast cancer^24–27^.

Drugs repurposed as mechanotherapeutics have already reached the clinic and are likely to be added to the treatment regimen of patients suffering from stiff, desmoplastic tumors^16^. Indeed, in a phase II trial, the anti-hypertensive drug losartan increased the fraction of pancreatic cancer patients undergoing successful surgical resections after chemoradiation^28^ and is now under clinical investigation with chemoradiation and immunotherapy (clinicaltrials.gov identifier NCT03563248). However, these mechanotherapeutic agents are subject to systemic adverse effects, which sets dosage limitations. Adverse effects might include hypotension for losartan, liver and kidney damage for tranilast and immunosuppression for dexamethasone. Incorporation of these agents into nanoparticle formulations could prevent adverse effects by reducing drastically the dose of the mechanotherapeutic administered—owing to improved pharmacokinetic properties and selective intratumoral accumulation and avoiding renal clearance. Recent reports indicated that angiotensin receptor blockers loaded in polymeric or liposomal formulations have shown to reprogram the TME and improve immunotherapy and nanomedicine^29,30^.

In this work, taking advantage of the mechanotherapeutic properties of tranilast, we developed tranilast loaded polymeric micelles which induce TME normalization more efficiently than the free drug and at a much lower dose as well as while improving the efficacy of nano-immunotherapy.

## Results

### Characterization of tranilast micelles and ability to reduce fibrosis signaling in human and murine CAFs

**Fig. 1:**
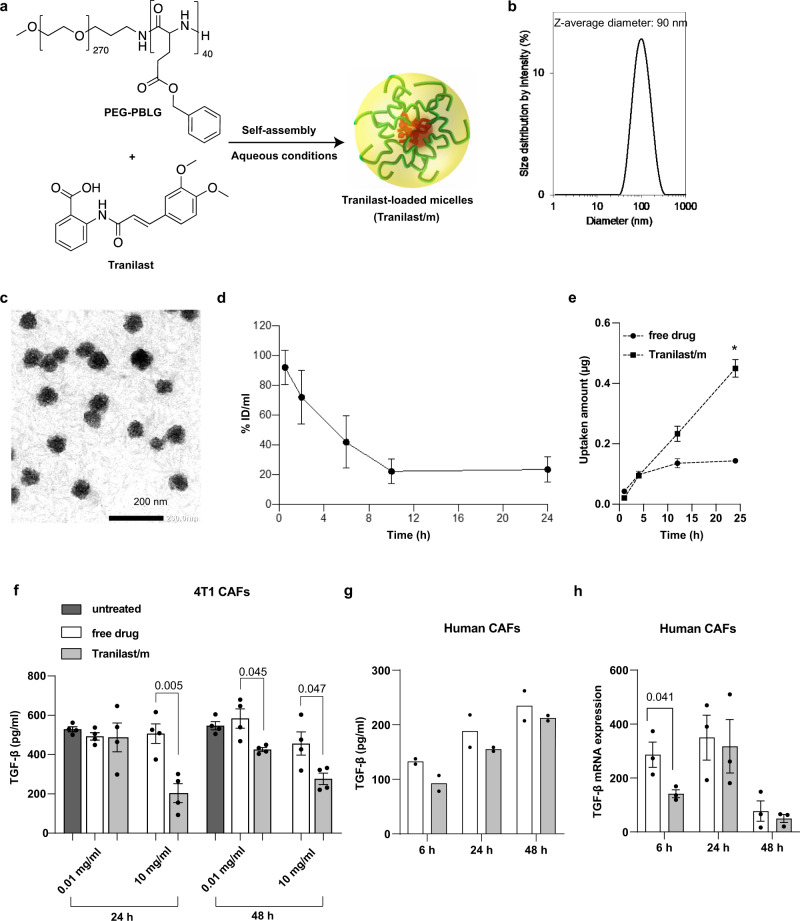
Characterization of tranilast-loaded micelles. **a** Schematic of tranilast-loaded micelles (Tranilast/m) preparation. The micelles were self-assembled by mixing PEG-PBLG and tranilast in aqueous conditions. **b** Size distribution of Tranilast/m determined by dynamic light scattering (DLS). **c** Transmission electron microscopy observation. **d** Time-dependent decay of plasma concentration after intravenous injection of 2 mg/kg Tranilast/m on tranilast basis. Data shown as the mean ± SD (*n* = 4 mice). **e** Tranilast uptake by 4T1-derived CAFs over time measured by HPLC. Data shown as the mean ± SD (*n* = 3 independent samples). **f** Quantification of TGF-β expression in conditional medium secreted by CAFs isolated from 4T1 breast tumors, following incubation with free tranilast or PEG-PBLG tranilast loaded micelles at a concentration of 0.01 and 10 mg/ml for 24 h and 48 h. Data shown as the mean ± SD (*n* = 8 independent samples for untreated group, *n* = 4 independent samples for treated group). **g** TGF-β1 protein concentration in conditional medium secreted by CAFs isolated from patients with lung adenocarcinoma (LUAD) (*n* = 2 independent samples). Data are presented as individual values and the mean. **h** mRNA expression levels of TGF-β by qPCR using GAPDH as the normalizing control (*n* = 3 independent samples). Data are presented as mean ± SE. Statistical analyses were performed by comparing the means of free drug and Tranilast/m samples using unpaired *t* test. TGF-β mRNA and protein was quantified following incubation with free tranilast or PEG-PBLG tranilast loaded micelles at a concentration of 0.01 mg/ml for 6, 24 and 48 h. Three replicates per sample were used and two independent experiments were performed. Data are presented as mean ± SE. Statistical analyses were performed by comparing means between two independent groups using the unpaired parametric Welch *t* test. Figure 1a was created with BioRender.com.

Tranilast loaded micelles were prepared by mixing PEG-*b*-poly (benzyl-l-glutamate) copolymer (PEG-PBLG) (Mw of PEG: 12,000; degree of polymerization of PBLG block: 40) and tranilast in tedrahydrofuran (THF) (Fig. 1a). The THF in the mixture was then evaporated using a rotatory evaporator, and the polymer and tranilast were resuspended using MilliQ water to form tranilast-loaded micelles (Tranilast/m). The micelles were sonicated and passed through a 0.22 μm filter. The free tranilast that was not loaded in the micelles was removed by dialysis against water. The micelles exhibited minimal batch-to-batch variation with an average size ~ 95 nm and polydispersity index values close to 0.12, as assessed by dynamic light scattering^31^ (Fig. 1b). Also, similar drug loading efficiency and drug/micelle ratio were observed between the different batches (Supplementary Fig. 4a, b). The Z-potential of the micelles at pH 7.5 was in the negative range but close to neutral (−0.74 mV). The loading of tranilast in the micelles was calculated to be 10% in weight of tranilast/weight of PEG-PBLG ratio using the UV absorbance of tranilast at 340 nm. Further characterization of micelles indicated high stability over the period of four weeks at 4 °C with size and polydispersity index values resembling the original values and without showing any precipitation in the solution (Supplementary Fig. 4c). The pertinent stability of size was also confirmed after dilution to a lower concentration in both PBS and 50% serum conditions (Supplementary Fig. 4d), further supporting their stability in physiological environments. Next, we sought to examine the critical micelle concentration (i.e., the minimum concentration required to maintain the micellar structure) by measuring the scattering light intensity. We found that Tranilast/m followed a linear drop until the concentration of 8 μg/ml (on micelle basis). At lower concentrations the slope of the count rate vs. micelle concentration changes (Supplementary Fig. 4e). Having determined the critical micelle concentration, we investigated whether concentrations lower or beyond the 8 μg/ml could affect drug release. To this end, we studied tranilast release at three different concentrations (4, 8 and 32 μg/ml) by the dialysis method in PBS containing 20% serum at 37 °C. Notably, tranilast was rapidly released from the micelles when these were used at 4 μg/ml, while less than 10% drug release was observed at 32 μg/ml concentration after 24 h incubation (Supplementary Fig. 4f). These data indicate that concentrations beyond the critical micelle concentration limit drug release and are suitable for in vivo applications. The transmission electron microscopy observation of the micelles was done after staining with 1% uranyl acetate (Fig. 1c). Subsequently, we measured the half-life of the Tranilast/m in blood. Since micelles may not exhibit the same kinetics as the free drug, we assessed the plasma clearance profile of the tranilast contained in the micelles using HPLC (Fig. 1d). We measured the half-life of the α-phase to be around 5.3 h and the half-life of the β-phase to be around 21.2 h. Moreover, the Vd was calculated as 0.93 ml. The pharmacokinetics of free tranilast have been studied in a previous work^32^, showing that around 98% of the initial dose of free tranilast is cleared within 2 h after intravenous injection. Thus, our Tranilast/m significantly improve blood circulation and bioavailability of the drug.

Then, we sought to investigate the effectiveness of the PEG-PBLG micelle formulation to deliver tranilast and to inhibit TGF-β expression in CAFs compared to free tranilast. We isolated CAFs either from patients with lung adenocarcinoma or from murine breast tumors (4T1) and treated them at different incubation times with either free drug or Tranilast/m. Our findings indicated that Tranilast/m increased cellular uptake (Fig. 1e) and suppressed TGF-β levels more effectively than the free drug independently of the incubation time (Fig. 1f), in murine CAFs. ELISA and RT-qPCR also confirmed that TGF-β levels were the most reduced in human CAFs treated with Tranilast/m compared to the free drug (Fig. 1g, h).

### Daily administration of Tranilast/m optimizes TME mechano-modulation and tumor perfusion

**Fig. 2:**
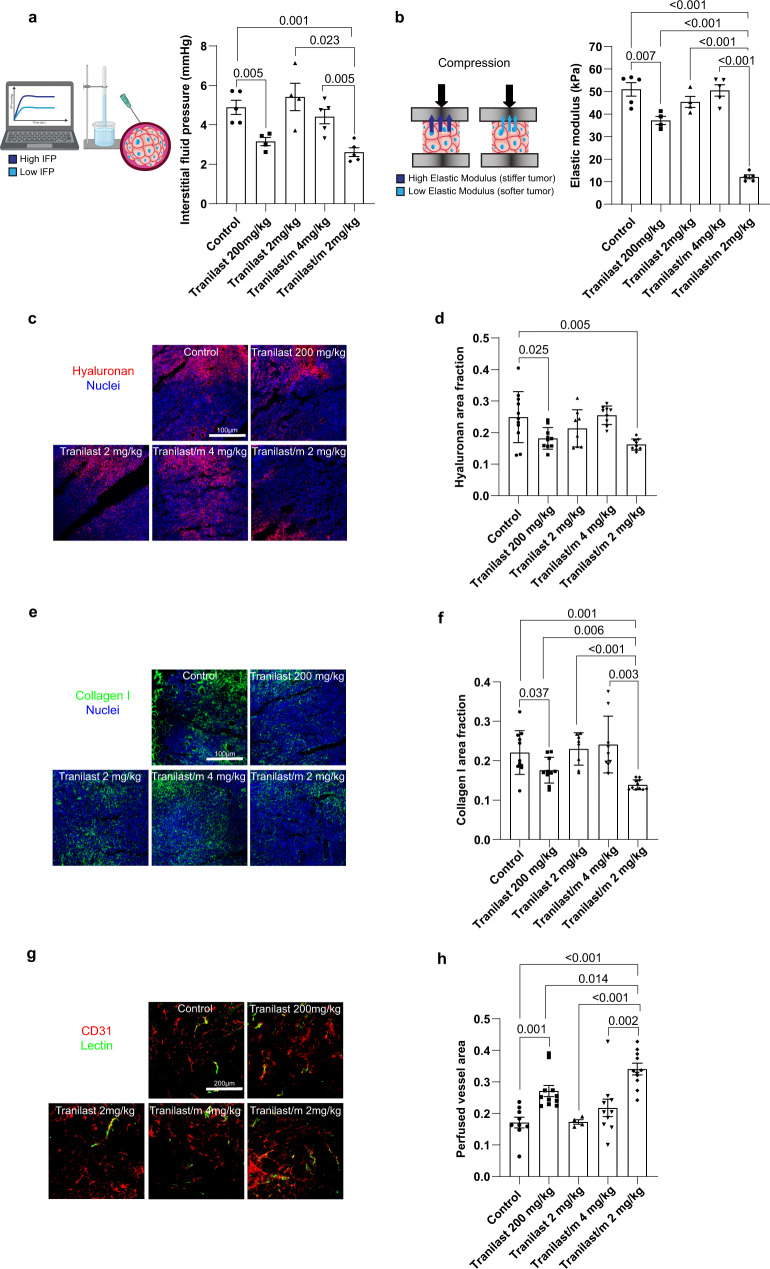
Normalization effects of free tranilast and Tranilast/m on the TME. **a** Quantification of interstitial fluid pressure (IFP) using the wick-in-needle technique in E0771 tumors treated with free tranilast 200 mg/kg (daily, orally), free tranilast 2 mg/kg (daily, i.v.), Tranilast/m 4 mg/kg (every other day, i.v.) and Tranilast/m 2 mg/kg (daily, i.v.) (*n* = 4–5 mice). **b** Ex vivo elastic modulus quantification of E0771 tumor specimens (3 × 2 × 2 mm, from the tumor interior) following unconfined compression to a final strain of 30% with a strain rate of 0.1 mm/min (*n* = 4–5 mice). **c** Representative fluorescence images of tumor tissue sections stained with anti-hyaluronan  (red) antibody and DAPI (blue). Scale bar 100 μm. **d** Quantification of the area fraction positive for hyaluronan staining treated as indicated (*n* = 3 mice, *N* = 3–4 image fields). **e** Representative fluorescence images of tumor tissue sections stained with anti-collagen I (green) antibody and DAPI (blue). Scale bar 100 μm. **f** Quantification of the area fraction positive for collagen I staining treated as indicated (*n* = 3 mice, *N* = 3–4 image fields). **g** Immunofluorescence staining of E0771 tumor tissues using the endothelial cell marker anti-CD31 (red) and biotinylated lectin (green) as a measure of vascular perfusion indicated by the colocalization of CD31 and lectin protein (yellow). Scale bar 200 μm. **h** Perfused vessel fraction in the different treatment groups normalized to total CD31 positive staining (*n* = 3 mice, *N* = 3–4 image fields). Data are presented as mean ± SE. For (**a**, **b**) (right panels), (**d**, **f**, **h**) statistical analyses were performed by comparing means between two independent groups using the unpaired parametric Welch *t* test. (**a**, **b**) were created with BioRender.com.

Next, we set out to test our hypothesis that Tranilast/m can potentiate normalization of the tumor stroma when administered in amounts significantly lower than those of the free agent. Mice-bearing syngeneic, orthotopic triple-negative breast tumors were treated either with free tranilast or the Tranilast/m. Free tranilast was administered at the optimal dose of 200 mg/kg orally every day^18,25^ or by intravenous injection (i.v.) at the dose of 2 mg/kg daily. Tranilast/m were administered i.v. following two protocols: a daily administration of 2 mg/kg or a 4 mg/kg dose every other day, so that at the end of the 6-day treatment the same dose of tranilast was administered through all i.v. protocols. Consistent with previous results none of the tranilast doses had any significant effect on tumor volume (Supplementary Fig. 5a). The effect of different tranilast treatments on normalizing the TME was first investigated in the context of physical properties, and in particular the interstitial fluid pressure and tissue stiffness. From the two Tranilast/m protocols, we found that only the daily 2 mg/kg dose managed to reduce fluid pressure and tumor elastic modulus as measured by the wick-in-needle technique and ex vivo stress-strain experiments, respectively (Fig. 2a, b). The decrease in these two physical properties of the tissue were significantly greater compared to the reduction caused by free tranilast administered orally at 200 mg/kg, whereas the intravenous injection of free tranilast had no effect. To further study changes in the elastic properties of the tumors, we monitored tumor stiffness during tranilast treatment with shear wave elastography (SWE) (Supplementary Fig. 5b). Before the start of tranilast treatment (day 12 post-implantation of cancer cells) tumors from all groups exhibited no change in the average elastic modulus, which was ~30 kPa. At the end of the treatment, however, the elastic modulus values measured with SWE were quantitatively similar to these obtained by the ex vivo stress-strain experiments. The group that received the 2 mg/kg Tranilast/m had a statistically significant reduction in the elastic modulus compared to all other groups. Atomic force microscopy and nanomechanical analysis were performed at the end of the experiment and confirmed the macroscopic observations that only the 200 mg/kg free drug and 2 mg/kg Tranilast/m had an effect on reducing tumor stiffness (Supplementary Fig. 5c).

Elevated interstitial fluid pressure and stiffness in desmoplastic tumors, such as the tumor models considered in our study, depend on large part on hyaluronan and collagen levels^33,34^. We assessed the levels of these key structural components using fluorescence immunostaining (Fig. 2c–f). Indeed, with found a decrease in hyaluronan and collagen I protein levels (Fig. 2d, f) and mRNA expression (Supplementary Fig. 5d). Significantly, we showed that Tranilast/m treatment suppressed collagen levels more effectively compared to free tranilast. Given these changes in tumor composition and stiffness, we reasoned that the abnormalities in vascular perfusion would be restored. Immunostaining of the endothelial cells using the anti-CD31 antibody and a streptavidin conjugate interacting with biotinylated Lycopersicon esculentum lectin, which was administered to perfuse animals prior to euthanasia, confirmed improved vascular perfusion (Fig. 2g, h). This enhanced vessel functionality was also associated with an increase in vessel diameter (Supplementary Fig. 5f), while no consistent change was noted in the total CD31 signal indicating that Tranilast/m exert their effect by facilitating vessel decompression rather than de novo formation of new vessels (Supplementary Fig. 5e). The increase in vessel diameter and perfused vessel area was statistically significant in the 2 mg/kg Tranilast/m compared to the free drug.

Finally, we aimed to provide an explanation for the finding that the higher, but less frequent dose of Tranilast/m, has no effect on modulating the TME compared to the lower and more frequent administration. We employed our previously developed mathematical model for nanoparticle delivery to tumors^35^ and simulated the experimental protocol of Tranilast/m administration. We found that after three cycles of treatment the intratumoral concentration of tranilast is more sustained and at higher levels in the case of the 2 mg/kg Tranilast/m compared to the 4 mg/kg Tranilast/m (Supplementary Fig. 5g), which could provide a reasonable explanation for the inability of the higher, less frequent dose to induce normalization effects.

We conclude that tranilast micelles at low, daily doses can more effectively cause normalization of the TME, reducing the required amount of tranilast administration by two orders of magnitude.

### Tranilast micelles enhance homogeneous accumulation and antitumor efficacy of cytotoxic nanomedicine and decrease lung metastases

Subsequently, we investigated the ability of Tranilast/m to improve the delivery and antitumor efficacy of nanoparticle formulations. In previous research, we found that intratumoral delivery of particles larger than 100 nm is challenging owing to vascular inefficiency and the dense extracellular matrix that inhibit the transport of the particles to the tumor site and their deep penetration into the tumor, respectively^36–38^. Here, we studied the delivery of 100 nm micelles using whole body fluorescence imaging. Specifically, BALB/c mice bearing 4T1 tumors were pre-treated with either Tranilast/m (2 mg/kg, daily) or control for 6 days and then received an intravenous injection of Cy5-labeled Tranilast-free micelles of the same composition. Tranilast/m treatment resulted in an increased and more homogeneous accumulation of micelles in the tumor site (Supplementary Fig. 6), underlining the significance of such normalizing agents in optimizing delivery of nanoformulations to desmoplastic tumors.

**Fig. 3:**
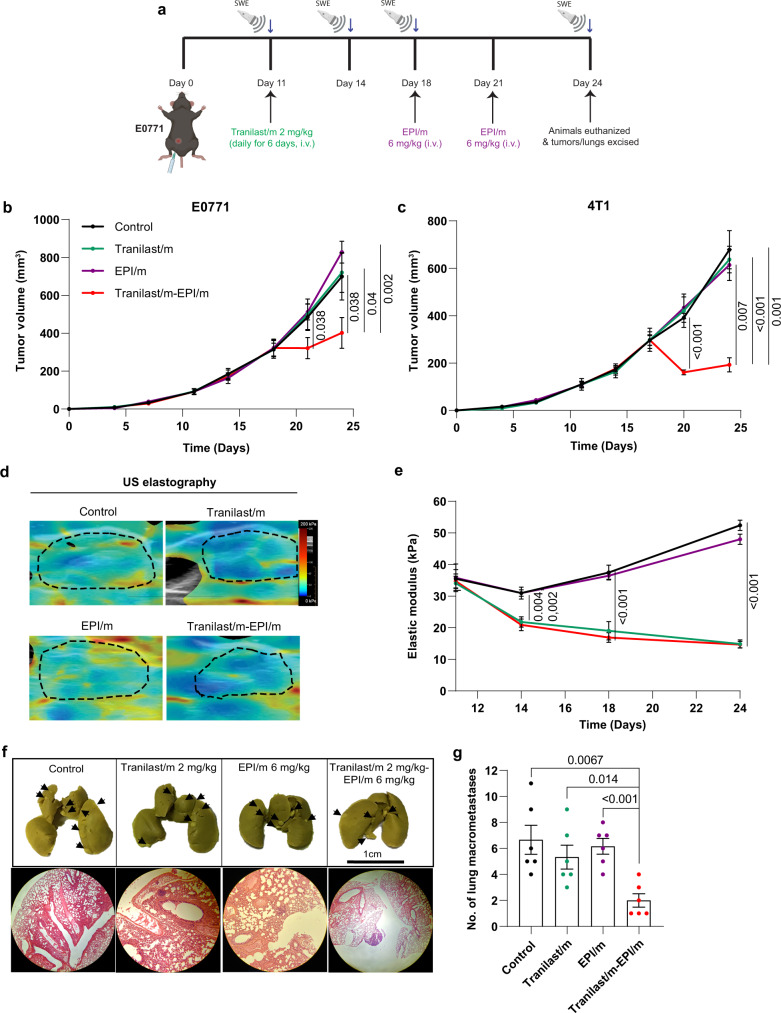
Tranilast micelles enhance efficacy of epirubicin micelles and decrease lung metastasis. **a** Study treatment protocol. Animals received daily intravenous injections of Tranilast/m 2 mg/kg for 6 days and subsequently two doses of EPI/m 6 mg/kg (on days 18 and 22 in E0771 tumors and on days 17 and 21 in 4T1 tumors). Orthotopic E0771 (**b**) and 4T1 (**c**) breast cancer primary tumor growth in mice treated with Tranilast/m, EPI/m or combination of the two. **d** Representative ultrasound elastography heat maps of E0771 tumors following different treatments, with blue indicating compliant tissue and red indicating stiff tissue. The dashed black line denotes the tumor margin (*n* = 4–5 mice, *N* = 2 image fields per mouse). **e** Elastic modulus values in E0771 tumors during treatment using ultrasound elastography. **f** Representative images of lungs of E0771 tumor bearing mice following fixation in Bouin’s solution and Hematoxylin and Eosin staining. Surface lung metastases are marked with black arrows. **g** Quantification of macroscopic E0771 spontaneous lung metastasis formation upon completion of the study. Data are presented as mean ± SE. Statistical analyses were performed by comparing means between two independent groups using the unpaired parametric Welch *t* test. (**a**) was created with BioRender.com.

Next, we studied the cytotoxic effects of 100 nm epirubicin loaded micelles (EPI/m) in combination with the Tranilast/m. EPI/m (also known as NC-6300) have demonstrated high efficacy and safety against various solid tumors in preclinical models and are currently evaluated in a phase I/II clinical trial (NCT03168061)^39^. Both 4T1 and E0771 orthotopic tumor models were employed and the treatment protocol is shown in Fig. 3a. Mice were treated daily with Tranilast/m or the control for six days and then two doses of 6 mg/kg EPI/m were administered. Tranilast/m-EPI/m combination suppressed primary tumor growth by more than 50% in both E0771 and 4T1 models, while monotherapies failed to induce any significant growth inhibitory effect (Fig. 3b, c and Supplementary Fig. 7a, b). Furthermore, using ultrasound elastography^22^, we confirmed in vivo the reduction in tumor stiffness caused by Tranilast/m treatment as the elastic modulus of E0771 and 4T1 tumors reduced significantly to 20 and 15 kPa, respectively. We also observed a 5–10% reduction in stiffness after EPI/m monotherapy, which is probably explained by the cytotoxic capacity of such anthracyclines to directly kill cells and reduce the intratumoral cell content (Fig. 3d, e, and Supplementary Fig. 7c, d). According to previous studies, EPI/m can directly target lymph node metastasis via the vasculature^40^, so we hypothesized that combining Tranilast/m with EPI/m could inhibit the formation of lung macrometastasis. We observed that the EPI/m monotherapy did not show any anti-metastatic effects, whereas its combination with Tranilast/m induced a remarkable low incidence in both E0771 and 4T1 tumor models (Fig. 3f, g and Supplementary Fig. 7e), without presenting significant toxicity as demonstrated by body, spleen and thymus weight (Supplementary Fig. 8). These observations suggest that normalization of the tumor microenvironment induced by the Tranilast/m facilitates EPI/m delivery and enhances antitumor efficacy in metastatic breast tumor models.

### Tranilast micelles improve the efficacy of nano-immunotherapy

**Fig. 4:**
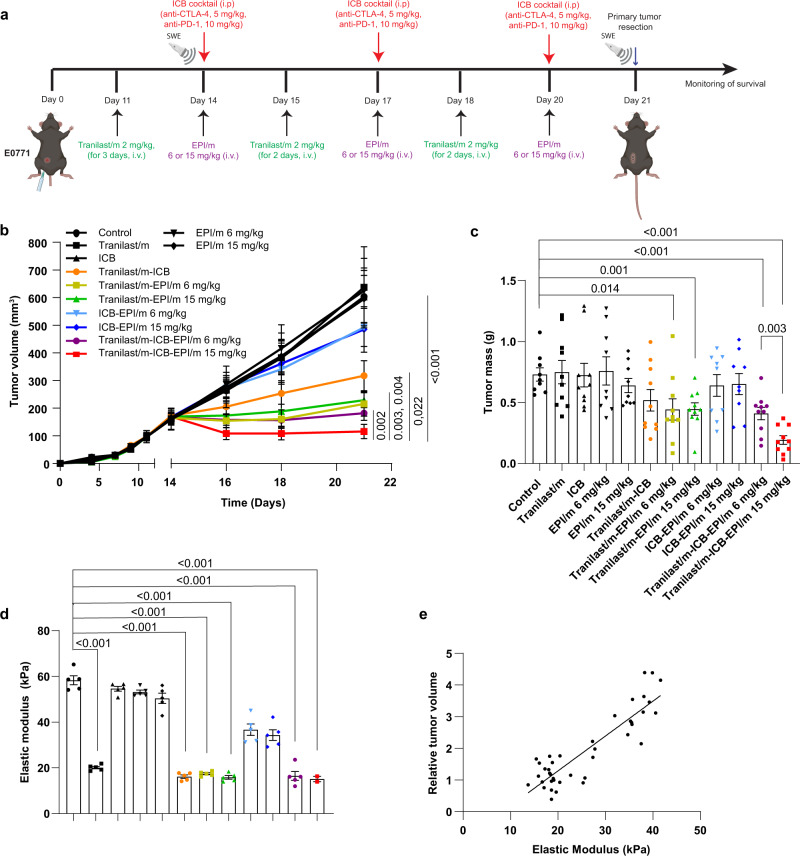
Tranilast micelles significantly improve the efficacy of nano-immunotherapy. **a** Study treatment protocol for the effect of tranilast micelles on nano-immunotherapy. Tumor volume (**b**) and mass (**c**) of E0771 tumors. Both 6 and 15 mg/kg of EPI/m chemotherapy exhibited a significant antitumor effect when combined with immunotherapy and tranilast treatment (*n* = 10 mice). **d** Tumor elastic modulus values measured with ultrasound elastography at the completion of the treatments (day 21). (*n* = 5 mice, *N* = 2 image fields per mouse). Data are presented as mean ± SE. For (**b**–**d**), statistical analyses were performed by comparing means between two independent groups using the unpaired parametric Welch *t* test. **e** Relative change in primary tumor volume at the end of the experiment compared to tumor volume prior to initiation of the treatments as a function of the elastic modulus before treatment initiation (*R*^2^ = 0.77, quantifiable analysis of how well the line of best fit, fits the data). (**a**) was created with BioRender.com.

Considering that the hypoxic microenvironment of many cancers is characterized by a poor immunogenicity allowing cancer cells to evade immune surveillance and attenuating the killing potential of immune cells^3,5,6,41^, we explored the antitumor efficacy of ICBs combined with epirubicin nanomedicine alongside with the tranilast micelles in orthotopic E0771 tumors. Similar to the neoadjuvant setting, animals were treated with control solution (anti-IgG, NaHCO_3_, 1x PBS), Tranilast/m (2 mg/kg), ICB cocktail (5 mg/kg anti-CTLA-4, 10 mg/kg anti-PD-1) and EPI/m at two different doses (6 or 15 mg/kg), either as monotherapy or combination therapy (Fig. 4a). Consistent with previous results, monotherapy administration provided insufficient antitumor responses as indicated by tumor growth and mass measurements. Combination of ICBs with EPI/m (6 or 15 mg/kg) or Tranilast/m exhibited a therapeutic effect corresponding to ~20 and 50% reduction in tumor volume, respectively, compared to the untreated control group (Fig. 4b). Co-administration of Tranilast/m with either dose of EPI/m induced a more potent antitumor activity, similar to that observed upon Tranilast/m-ICB-EPI/m at 6 mg/kg combination treatment (~65% tumor regression), which did not, however, outperformed the effect of maximum tolerated dose of EPI/m (15 mg/kg) eradicating 85% of tumor volume (Fig. 4b). The synergistic effects of Tranilast/m with ICB, EPI/m or the combination of the two was also obvious on the reduction of tumor mass (Fig. 4c). We further measured tumor stiffness with ultrasound elastography to confirm that changes in the efficacy of the ICBs and EPI/m are related to this physical parameter of the TME. All mice receiving Tranilast/m exhibited the same reduction in tissue stiffness corresponding to an elastic modulus of approximately 20 kPa. This reduction remained constant throughout the ICB and/or EPI-m treatment, further supporting our conclusion that neither ICB nor EPI-m alone can mechanically intervene in the supporting tumor stroma composition (Fig. 4d, Supplementary Fig. 9a). None animal exhibited significant weight loss after different treatments (Supplementary Fig. 9b).

Taking into consideration that EPI/m are currently under clinical evaluation, we investigated whether the combination of Doxil, which is an already approved nanomedicine for metastatic breast cancer, exhibits similar antitumor responses. Of note, we found that Tranilast/m potentiate both ICB and Doxil antitumor activity as demonstrated by the reduction in tumor volume (Supplementary Fig. 10a–c), and also combination of either Doxil or EPI/m with Tranilast/m eliminated tumor volume by 67% compared to the untreated control of each study.

Monitoring tissue stiffness with ultrasound elastography, we confirmed the changes in the mechanical properties of tumor during treatment (Supplementary Fig. 10d). Based on these results, we hypothesized that the elastic modulus of tumor correlates with response to therapy and thus, making tissue stiffness a potential mechanical biomarker for therapy prediction. To explore this, we plotted the relative change in tumor volume at the end of the various therapies compared to the tumor volume prior to the initiation of the treatments as a function of the elastic modulus of the tumors measured before treatments initiation. Even when we consider the data from the epirubicin micelles study alone (Fig. 4e) or the results of both the epirubicin micelles and Doxil studies together (Supplementary Fig. 10e), there is a linear correlation of tumor stiffness with the efficacy of any therapy, either nanomedicine (EPI/m and Doxil) or immunotherapy or combination of the two.

Finally, we examined whether tranilast treatment has a direct effect on CAFs activity, which could cause the reported reduction in tumor stiffness through remodeling of the extracellular matrix. Using tumor sections, we labeled CAFs with alpha smooth muscle actin (αSMA), a protein highly expressed in myofibroblasts in the tumor microenvironment and the proliferation marker Ki67. Indeed, our findings indicate that Tranilast/m alone or combination therapy decreases both CAF population and proliferation (Supplementary Fig. 11).

### Combining tranilast micelles with cytotoxic nanomedicine enhances T cells infiltration

**Fig. 5:**
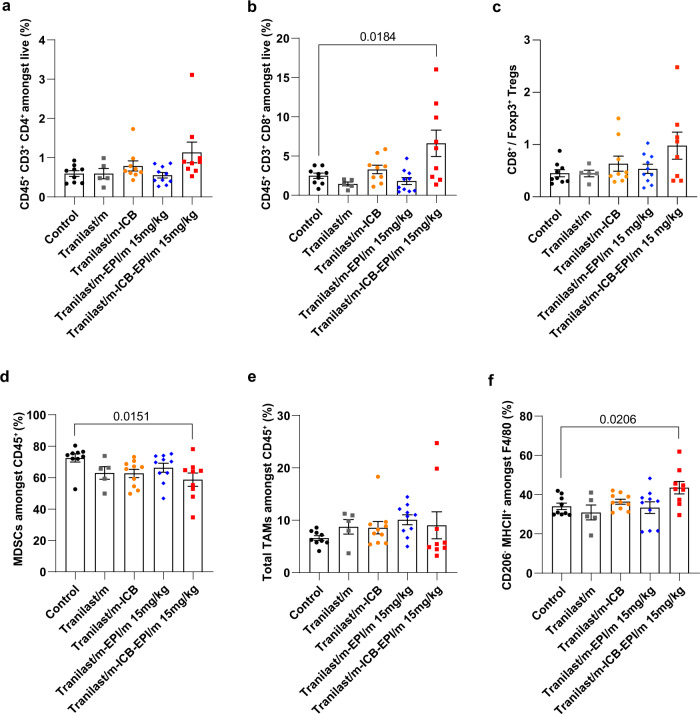
Tranilast micelles combined with nano-immunotherapy enhance tumor immunogenicity. **a** Percentages of intratumoral CD45^+^ CD3^+^ CD4^+^ (SP, single positive) cell population and (**b**) percentages of intratumoral CD45^+^ CD3^+^ CD8^+^ (SP, single positive) cell population gated on live cells in different treatment groups, measured by flow cytometry (n = 5-9 mice). (**c**) Ratio of cytotoxic CD8^+^ T cells to immunosuppressive CD4^+^ regulatory T cells (Tregs) significantly increases after Tranilast/m-ICB-EPI/m combination treatment (*n* = 5–9 mice). Tregs are defined as Foxp3^+^CD127^lo^CD25^hi^ CD4 SP gated on CD45^+^ lymphocytes. **d** Percentages of intratumoral MDSCs (CD45^+^ CD11b^+^ GR1^+^) and (**e**) TAMs (CD45^+^ CD11b^+^ GR1^-^ F4/80^+^) gated on CD45 lymphocytes (*n* = 5–9 mice). **f** Percentage of antitumor M1-like TAMs (CD45^+^ CD11b^+^ GR1^-^ F4/80^+^CD206^-^MCHII^+^) gated on total TAM population. M1-like murine macrophages are characterized by enhanced expression of MHC class II and downregulation of mannose receptor CD206. Data are presented as mean ± SE. Statistical analyses were performed by comparing means between two independent groups using the unpaired parametric Welch t-test.

Given that vascular functionality and tissue oxygenation relate to increased immune cell infiltration and activity^25^, we sought to examine whether the potent antitumor responses of EPI/m at 15 mg/kg combined with immunotherapy in normalized tumors relies on the level of tumor immunogenicity. Flow cytometry analysis showed that priming of the tumor microenvironment with tranilast enhanced the antitumor responses of immunotherapy as indicated by the accumulation of CD4^+^ and CD8^+^ T cells and the concomitant increase in the cytotoxic to regulatory T cell ratio (Fig. 5, Supplementary Fig. 12). Notably, this effect on immunostimulation becomes stronger upon epirubicin co-administration indicating the potential of such combination treatments in reverting the “cold”, immune-excluded phenotype of control tumors into highly immunogenic. Accordingly, the normalization effects of the tranilast micelles could be used not only as a strategy to optimize targeted delivery of cytotoxic nanomedicines, but also to block the immunosuppressive tumor bed in order to enhance tumor sensitization to immunotherapy, which is supported by the reduction of myeloid-derived suppressor cells (MDSCs) (Fig. 5d). On the other hand, levels of tumor associated macrophages (TAMs) remain the same upon different treatments, while their specific polarization into the immunostimulatory M1-like subpopulation is increased in Tranilast/m-ICB-EPI/m 15 mg/kg treated mice (Fig. 5e, f).

### Tranilast-epirubicin micellar combination increases overall survival and induces immunological memory

**Fig. 6:**
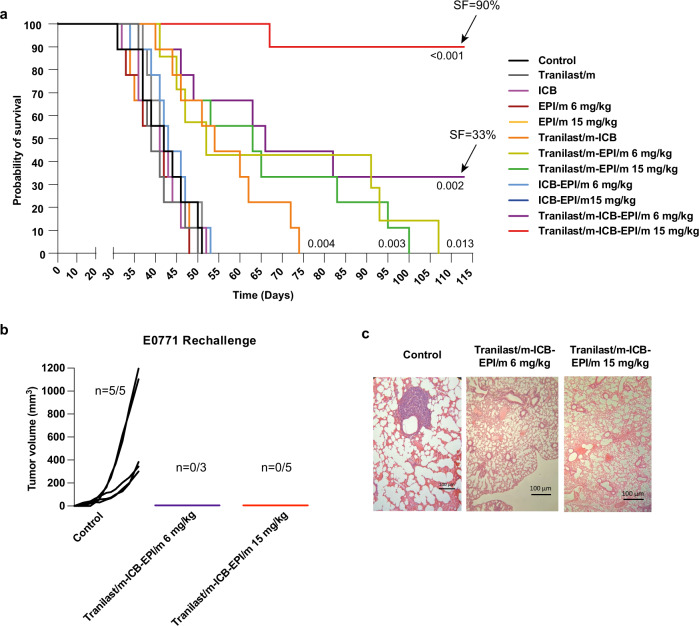
Tranilast-epirubicin micellar combination increases overall survival, potentiating immunological memory. **a** Kaplan–Meier survival curves for the various treatments considered (*n* = 10 mice). Arrows indicate the survival fraction (SF) of mice at day 113. Statistical analysis was performed by a log-rank (Mentel-Cox) test comparing the treated groups with the control. **b** Individual tumor growth curves of the surviving E0771 bearing mice rechallenged with E0771 cancer cells versus control mice naïve to E0771 cells inoculated on day 0 (*n* = 3–5 mice). **c** Lungs excised from mice that were rechallenged with E0771 cells at the study endpoint and assessed for tissue micrometastasis via H&E (*n* = 5 control, 3 Tranilast/m-EPI/m mice and 5 Tranilast/m-ICB-EPI/m mice).

The primary tumor growth and immunogenicity data indicated that combining cytotoxic nanomedicine with immunotherapy in normalized tumors promotes restoration of the antitumor immune defense. To test whether this synergy positively correlates with survival outcome, we surgically excised primary tumors after completion of the treatment protocol aiming to assess mice survival against spontaneous metastases that potentially arose during treatment. We found that combined treatment with Tranilast/m, EPI/m at 15 mg/kg and the ICB cocktail significantly extended the survival of mice, with 9 out of 10 animals being alive for 113 days, while the lower dose of EPI/m (6 mg/kg) improved survival to a lesser extent with 3 out of 10 survived (Fig. 6a). To verify the efficacy of combination treatment in inhibiting metastasis formation, we sacrificed 3 from the survived mice that received the high dose of epirubicin and found no evidence of surface macrometastases in the lungs (Supplementary Fig. 13). The remaining 3 and 6 mice of Tranilast/m-ICB-EPI/m 6 mg/kg and 15 mg/kg treatment groups, respectively, were rechallenged with a second inoculation of E0771 cells and their individual growth rates were compared against 5 healthy control mice of the same age. Remarkably, tumors developed in all control but none in the combination-treated mice that survived the initial experiment (Fig. 6b). Finally, animals were scarified 27-days post-rechallenge and lung metastasis was assessed by tissue staining with hematoxylin and eosin (Fig. 6c). Significantly, metastatic lesions were detected only in the control group, suggesting that mice achieved durable long-term antitumor responses and acquisition of immunological memory following treatment with epirubicin and ICB combination.

**Fig. 7:**
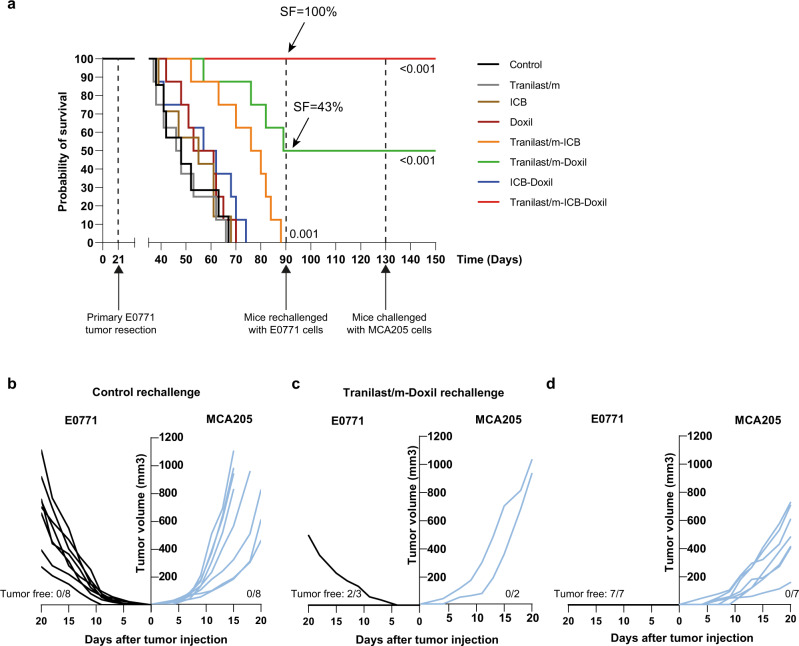
Specificity and longevity of antitumor response elicited by the combination of Tranilast/m with nano-immunotherapy. **a** Kaplan–Meier survival curves for the various treatments considered (*n* = 7–8 mice). Arrows indicate the survival fraction (SF) of mice at day 90. Statistical analysis was performed by a log-rank (Mentel-Cox) test comparing the treated groups with the control. C57BL/6 female mice were orthotopically injected with 5 × 10^4^ E0771 cells on day 0 and subsequently treated as indicated. Primary tumors were surgically removed on day 21 and mice were daily monitored for their survival rate. On day 90, survivors of the Tranilast/m-Doxil (*n* = 3) and Tranialst/m-ICB-Doxil (*n* = 7) group were rechallenged with 5 × 10^4^ E0771 cells in the opposite site of the first injection. A group of naïve mice of the same age was challenged in parallel to serve as a control. On day 130, tumor free mice and a group of naïve mice were challenged in parallel with the irrelevant MCA205 fibrosarcoma cell line (2.5 × 10^5^ cells) by subcutaneous injection in the right flank. **b**–**d** Individual growth curves of naïve mice (**b**), cured mice of Tranilast/m-Doxil group (**c**) and Tranilast/m-ICB-Doxil group (**d**) challenged with E0771 (left) and MCA205 (right) tumor cells. The number of tumor free mice is also demonstrated in each study.

To further support our hypothesis of specificity and longevity of antitumor response elicited by the combination of Tranilast/m with nano-immunotherapy, we employed an independent survival study where we replaced the epirubicin treatment with Doxil. For this study, mice were orthotopically injected with 5 × 10^4^ E0771 cells on day 0 and treated following the same treatment protocol as before (Supplementary Fig. 3). Primary tumors were removed on day 21 and animals were monitored for their survival rate. Treatment with Tranilast/m, ICB and Doxil monotherapies as well as ICB-Doxil combination failed to provide any survival advantage or tumor regression compared to control group (Fig. 7a). Treatment with Tranilast/m-ICB provided a significant benefit, but not strong enough for mice to attain complete response. On the contrary, combination of Tranilast/m with Doxil yield to a more potent effect, with half of mice exhibiting a complete response, while treatment with all three drugs yield to a 100% survival. On day 90, all surviving mice from the Tranilast/m-Doxil and Tranilast/m-Doxil-ICB combination groups, along with a group of eight naïve mice were challenged with E0771 cells. As expected, all naïve mice succumbed. Two out of three mice of the Tranilast/m-Doxil group that survived the first E0771 challenge were resistant to rechallenge, while all mice of the triple combination treatment group successfully rejected E0771 cells. Tumor-free mice were challenged again on day 130 with a subcutaneous injection of 2.5 × 10^5^ cells from the unrelated MCA205 fibrosarcoma tumor line. A group of naïve mice was challenged in parallel. Notably, none of the mice in either group was able to reject this irrelevant tumor line, indicating that the long-term immune memory acquired is tumor specific (Fig. 7b-d).

## Discussion

There is a growing appreciation of the role of the physical microenvironment in cancer, which has led to several discoveries about the origins and consequences of the physical traits of cancer^8,34,42^. These discoveries have resulted in new targets and treatment strategies that involve the modulation of the mechanical TME with the use of specific mechanotherapeutics prior to cytotoxic therapy^5,16^. The first successful clinical trial of losartan in combination with chemoradiation in locally advanced pancreatic cancer patients has highlighted the promise of this therapeutic strategy to improve cancer therapies^16,28^. Scientific research should now focus on the optimized use of mechanotherapeutics, which involves monitoring of mechanical aspects of the TME for patient-specific treatment and development of predictive biomarkers for optimized use of mechanotherapeutics and therapeutic strategies.

To this direction, we developed a micellar nanoformulation of the mechanotherapeutic tranilast. Employing two syngeneic triple negative breast tumor models (4T1 and E0771), tranilast micelles were found to be more effective than free tranilast when administered in a dose 100 times lower than that of the free drug. The micellar formulation of tranilast was also found to boost the delivery and efficacy of epirubicin micelles and Doxil, overcome immune checkpoint resistance, induce tumor immunogenicity and improve the efficacy of nano-immunotherapy leading to cure.

Tranilast induces leukopenia^43^, which predicts poor immune checkpoint inhibition outcome. Combining tranilast with chemotherapy, which also induces leukopenia, could further reduce immune checkpoint inhibition efficacy. Thus, developing tumor normalizing treatment strategies for chemo-immunotherapy that limit systemic effects is an urgent medical need. Nanocarriers can reduce toxicity induced by free drugs through selective accumulation in tumors^44^, even though they might be associated with other adverse effects. Here, we developed micelle-loaded tranilast (Tranilast/m) using PEG-b-poly (benzyl-L-glutamate) copolymer and tested whether it can reduce the dose required for normalization. In addition, we combined the tranilast micelles with a pH-sensitive micelle formulation of epirubicin chemotherapy (EPI/m), which itself limits neutropenia^45,46^. Unexpectedly, we found the tranilast-loaded micelles to induce improved normalization effects in breast tumor models compared to the free tranilast, enabling dose reduction by 100-fold. Tranilast is a small molecule with an estimated blood half-life in humans of approximately 5 h^47^, which in mice is expected to be much less. With the incorporation of tranilast into micelles, we managed to increase significantly the half-life of the terminal elimination phase to 21.2 h, improving considerably its pharmacokinetic properties. The micelles were capable of normalizing the mechanical tumor microenvironment and also enhanced epirubicin micelles accumulation, achieved homogenous intratumor nanoparticle microdistribution, and increased cancer-associated fibroblast uptake of tranilast leading to reduced TGF-β signaling. Tranilast micelles were found to inhibit pro-tumor immunosuppressive signaling and increase T cell infiltration in immune “cold” solid tumors, indicating that TGF-β targeting is a promising strategy for overcoming immune checkpoint blockade therapy resistance. It should be further mentioned that ICBs are macromolecules (antibodies) and their penetration into the tumor is also impaired by stiffness, so that as with nanomedicines they will also penetrate tumors better when stiffness is reduced. Importantly, combination of tranilast micelles with nano-immunotherapy led to complete cure in 9 out of the 10 mice that received the highest dose of epirubicin micelles and in 7 out of 7 mice that received Doxil. These results highlight the high potential of mechanotherapeutics to improve the efficacy of nano-immunotherapy.

In addition, our study showed the applicability of the clinically applied ultrasound shear wave elastography to monitor the effects of the mechanotherapeutic agent on tumor stiffness and also to predict the therapeutic outcome of nanomedicine, immunotherapy and the combination of the two, similar to our previous findings^22^. Furthermore, apart from ultrasound, magnetic resonance elastography can quantify tumor stiffness with a focus on brain tumors^48^. Therefore, we propose that tumor stiffness measured with established imaging techniques should be considered as an immune-predictor and should be further investigated as potential biomarkers of response to nano- and immuno-therapies and for monitoring the efficacy of mechanotherapeutic treatments.

## Methods

### Cell culture

4T1 (ATCC CRL-2539) and E0771 (94A001, CH3 BioSystems) mouse breast adenocarcinoma cell lines were purchased form ATCC and CH3 BioSystems, respectively. The cells were maintained at 37 °C/ 5% CO_2_ in Roswell Park Memorial Institute medium (RPMI-1640, LM-R1637, biosera) supplemented with 10% fetal bovine serum (FBS, FB-1001H, biosera) and 1% antibiotics (A5955, Sigma). The MCA205, mouse fibrosarcoma cell line was purchased from Millipore (SCC173, Millipore) and cultured in expansion medium consisting of RPMI-1640 (LM-R1637, biosera) supplemented with 2 mM L-glutamine (TMS-002-C, Sigma), 1 mM sodium pyruvate (TMS-005-C, Sigma), 10 % fetal bovine serum (FBS, FB-1001H, biosera), 1x non-essential amino acids (TMS-001-C, Sigma), 1% antibiotics (A5955, Sigma) and 1x β-mercaptoethanol (ES-007-E, Sigma). MCA205 cells were also maintained at 37 °C/ 5 % CO_2_.

### Drugs and reagents

Epirubicin micelles (EPI/m) were prepared as described in our previous report^49^. Briefly, a methanolic solution of PEG-poly(aspartate-hydrazide-epirubicin) was evaporated in a round-bottom flask. The resulting thin film was solvated by HEPES and sonicated to produce EPI/m. The size of EPI/m was determined by DLS using Zetasizer Nano ZS (Malvern Instruments, UK). The micelles were purified using a centrifugal filter unit (molecular weight cut-off (MWCO): 30,000 Da). The drug loading was evaluated by HPLC after completely cleaving the drug from the polymer by adding 1 N HCl. The loading was calculated as 0.15 mg of epirubicin per 1 mg of micelle.

Tranilast (Rizaben, Kissei Pharmaceutical, Japan) was dissolved in 1% NaHCO_3_ (33.3 mg/ml) following incubation at 70 °C for an hour, as previously described^18^. The ICBs mouse monoclonal PD-1 (CD279, clone RMP1-14) and mouse monoclonal CTLA-4 (CD152, clone 9D9) were purchased from BioXCell.

Tranilast/m were prepared by mixing 100 mg PEG-*b*-poly(benzyl-l-glutamate) copolymer (PEG-PBLG) (Mw of PEG: 12,000; degree of polymerization of PBLG block: 40) and 25 mg tranilast in THF (50 ml). The THF in the mixture was then evaporated by using a rotatory evaporator, and the polymer and tranilast were resuspended by using MilliQ water (100 ml) to form Tranilast/m. The micelles were sonicated and filtered by using a 0.22 μm filter. The micelles were then purified by dialysis against MilliQ (MWCO of the membrane: 3,000 Da). The diameter of Tranilast/m was assessed by DLS. To measure the Z-potential of the micelles, the micelle concentration was fixed at 0.5 mg/ml by diluting in 10 mM phosphate buffer (pH 7.4) and the surface charge was measured by using Zetasizer Nano ZS with folded capillary zeta cell. The drug loading of tranilast in the micelles was measure by freeze drying 1 ml of micelles to calculate the total micelle weight per ml, followed by redissolving the sample in acetonitrile and assessing the UV absorbance of tranilast at 340 nm with a UV-spectrometer (JASCO, Japan). To assess the batch-to-batch variability, three different batches of Tranilast/m were prepared and characterized by the same method described above. The long-storage stability of micelles was determined by diluting Tranilast/m in water to a final concentration of 1 mg/ml and maintained at 4 °C for a month. The size distribution of the micelles was evaluated every week by DLS measurement. Next, stability of micelles was assessed under isotonic conditions (PBS) and 50% serum to mimic the physiological environment during circulation. Micelles were diluted in either PBS or PBS containing 50% FBS to a final concentration of 0.1 mg/ml on micelle basis. The samples were kept at 37 °C like the body temperature. The size of the Tranilast/m was monitored by dynamic light scattering measurement at different time points for 24 h to evaluate the stability as described before. The critical micelle concentration (CMC) of Tranilast/m was determined by light scattering measurement following the reported protocol^31^. Briefly, Tranilast/m (1 mg/ml on a micelle basis in pure water) were sequentially diluted in pure water to concentrations ranging from 1–64 μg/ml on a micelle basis. The samples were loaded to Zetasizer (Malvern Instruments Limited, U.K.) equipped with 532 nm laser and the scattered light was detected at an angle of 173°. Additional characterization of drug release profile was performed by dialysis method. Specifically, Tranilast/m (1 mg/ml on micelle basis) were diluted in 1 ml PBS (pH 7.4) containing 20% serum to final concentrations below the CMC (4 μg/ml), near the CMC (8 μg/ml) and above the CMC (32 μg/ml). The samples were then loaded in a dialysis bag (MWCO: 10,000 Da) and dialyzed against 99 ml PBS (pH 7.4) containing 20% serum at 37 °C. At determined time points (2, 4, 8 and 24 h), 100 μl were sampled from the micelle solution inside the dialysis bag and mixed with 400 μl of DMSO. Then, the collected sample was filtered through a 0.45 μm filter. The filtered samples were then loaded into an HPLC system (column: TSKgel ODS-100V 5 μm; mobile phase: DMF; detector: UV absorbance at 340 nm) to determine the tranilast concentration.

### Syngeneic tumor models and treatment protocols

All orthotopic models for murine mammary tumors were generated by implantation of 5 × 10^4^ 4T1 or E0771 cancer cells in 40 μl of serum-free medium into the third mammary fat pad of 8-week-old BALB/cOlaHsd and C57BL/6OlaHsd female mice, respectively. All mice were maintained in specific pathogen-free conditions in the animal facilities of Cyprus Institute of Neurology and Genetics. They were housed in controlled temperature/humidity (22 °C/55%) environment on a 12-h light-dark cycle and kept with free access to food and water throughout the whole experiment period. All in vivo experiments were conducted in accordance with the animal welfare regulations and guidelines of the Republic of Cyprus and the European Union (European Directive 2010/63/EE and Cyprus Legislation for the protection and welfare of animals, Laws 1994–2013) under a license acquired and approved (No CY/EXP/PR.L2/2018, CY/EXP/PR.L14/2019, CY/EXP/PR.L15/2019, CY/EXP/PR.L03/2020) by the Cyprus Veterinary Services committee, the Cyprus national authority for monitoring animal research for all academic institutions.

The inhibitory effect of each treatment on primary tumor growth was monitored by tumor volume. Planar dimensions (*x*, *y*) of tumor were measured every 2–3 days using a digital caliper and tumor volume was estimated from the volume of an ellipsoid and assuming that the third dimension, *z*, is equal to *xy*. The maximal tumor burden of 1200 mm^3^, as approved by the ethics committee, was not exceeded.

### Statistical analysis

Statistical analysis was performed using GraphPad Prism 8 software. The *p*-values and statistical tests performed are indicated in the figure and associated legend, respectively. A *p*-value of less than or equal to 0.05 was considered statistically significant.

### Reporting summary

Further information on research design is available in the Nature Portfolio Reporting Summary linked to this article.

## Supplementary information

Supplementary Information

Reporting Summary

## Data Availability

All data that support the findings of this study are available within the article and the supplementary information. Source data are provided with this paper.

## Source data

Source Data

## Code availability

MATLAB code used for image analysis is available at Zenodo https://zenodo.org/record/7151427#.Y1ovY3bMKUk^50^.
