# Supplementary material for: Polymeric micelles effectively reprogram the tumor microenvironment to potentiate nano-immunotherapy in mouse breast cancer models
Source: Nat Commun. 2022 Nov 22;13:7165. doi: 10.1038/s41467-022-34744-1 (PMC9684407; doi:10.1038/s41467-022-34744-1)
Supplement: Supplementary file 1 — Supplementary Information [file 41467_2022_34744_MOESM1_ESM.docx]

**Polymeric micelles effectively reprogram tumor microenvironment to potentiate nano-immunotherapy in mouse breast cancer models**

**Supplementary Information**

**Supplementary Methods**

Primary culture of CAFs

CAFs were obtained from surgically resected lung adenocarcinoma patients at National Cancer Center Hospital East. Tumors with a volume of 5 mm^3^ were cut into pieces and plated onto a 10 cm dish containing 2 ml of Minimum Essential Medium alpha (alpha MEM) with 10% fetal bovine serum (FBS) and 1% penicillin and streptomycin. The tissues were removed after they were surrounded by adherent fibroblasts, and the fibroblasts were cultured for more days. When the fibroblasts reached 80% confluency, they were harvested and re-plated at a density of 1x10^4^ cells/cm^2^. CAFs were incubated at 37 °C in an atmosphere containing 5% CO_2_. Experiments using human materials were approved by National Cancer Center Institutional Review Board (2005-043).

In vitro tranilast treatment

Adenocarcinoma was established and used for in vitro assay. ELISA assay. 8x10^4^ CAFs were maintained in 1 ml alpha MEM supplemented with 10 % FBS in a 24-well plate at 37 °C/ 5 % CO_2,_ for 24 h. Following culture media aspiration, CAFs were incubated with 1 ml of either free tranilast or PEG-PBLG micelles diluted in serum free culture media solution at a final concentration of 0.01 mg/ml. Also, an untreated control condition (CAFs with no drug) was used for background calibration. Secreted TGF-β in culture supernatant was measured at 6, 24 and 48 h using the human TGF-β1 ELISA Kit (KE00002, proteintech), according to the manufacturer’s instructions.

RNA Isolation, gDNA removal, cDNA Synthesis, and Real-Time PCR. Total RNA was isolated from human CAFs or E0771 tumor tissue using NucleoSpin RNA Plus (MACHEREY-NAGEL), and gDNA removal and cDNA synthesis was performed using PrimeScript RT reagent Kit with gDNA Eraser (TaKaRa). Real-time polymerase chain reaction was carried out using TB Green Prime Ex TaqII (TaKaRa). Reactions were performed using Thermal Cycler Dice Real Time System III (TaKaRa) at the following conditions: 95 °C for 2 min, 95 °C for 2 sec, 60 °C for 20 sec, 60°C for 1 sec, steps 2–4 for 40 cycles. Real-time PCR analysis and calculation of changes in gene expression between groups was performed using the ΔΔ^Ct^ method. Relative gene expression was normalized based on the expression of GAPDH (for human gene expression) and β-actin (for mouse gene expression). For qPCR analysis, 3 biological samples were used per treatment and 2-3 technical replicates for each sample. The specific human primers used for gene expression analysis were as follows: TGF-β primers: forward 5’-TCCTGGCGATACCTCAGCAA-3’ and reverse 5’- GCTAAGGCGAAAGCCCTCAA -3’; GAPDH primers: forward 5’-GCACCGTCAAGGCTGAGAAC-3’ and reverse 5’-TGGTGAAGACGCCAGTGGA-3’. The specific mouse primers used for gene expression analysis were as follows: Col1A1: forward 5’-*GAGCGGAGAGTACTGGATCG*-3’, reverse 5’-*GTTCGGGCTGATGTACCAGT*-3’, HAS2: forward 5’-*ATAAGCGGTCCTCTGGGAAT*-3’, reverse 5’-*CCTGTTGGTAAGGTGCCTGT*-3’, HAS3: forward 5’-*TTCCAAACCTCAAGGTGGTC*-3’, reverse 5’-*TGCTACGCCACACAAAGAAG*-3’, B-actin: forward 5’-*GACGGCCAGGTCATCACTAT*-3’, reverse 5’-*AAGGAAGGCTGGAAAAGAGC*-3’.

Cellular uptake and TGF-β secretion from 4T1 CAFs

4T1 CAFs were isolated from primary murine 4T1 tumors. BALB/c female mice bearing orthotopic 4T1 tumors (average tumor volume ≈ 200 mm^3^) were sacrificed for harvesting tumors. The tumors were minced and digested in Dulbecco's Modified Eagle Medium (DMEM) medium containing 0.1% type I collagenase, 10% FBS and 1% penicillin and streptomycin for 3 hours at 37 ℃. Cell suspensions were filtered through 100 μm strainer and centrifuged. The pellets were then resuspended in complete RPMI1640 medium and cultured in a flask. The cells attaching to the flask bottom within 20 min were recognized as CAFs and the floating cells were abandoned. This isolation process was repeated for 3 times to get purified CAFs. For detecting the cellular uptake of tranilast or Tranilast/m by CAFs, CAFs were seeded in 24 well plates (10^6^ cells in 1 ml medium per well) and incubated with tranilast or Tranilast/m (10 μg/ml tranilast equivalence). At determined time points, the culture medium was removed, and the cells were collected in 1 ml DMSO by scraping. The suspension samples were sonicated in 37 ℃ for 30 min, then centrifuged (10000 g x 10 min) to move the debris and loaded to HPLC system (solid phase: TSKgel ODS-100V 5 μm; mobile phase: DMF; detector: UV absorbance 340 nm). For measuring TGF-β secretion, CAFs were seeded to 96 well plate (2x10^4^ cells in 100 μl medium per well) and incubated with tranilast or Tranilast/m (10, 0.1 mg/ml tranilast equivalence). After 24 or 48 h incubation, the culture supernatants were collected for measuring TGF-β concentration by ELISA kit.

Syngeneic tumor models and treatment protocols

Tranilast dose response studies. C57BL/6 female mice having orthotopic E0771 tumor were allowed to reach a volume of 100 mm^3^ and randomized to five groups before treatment (n=5 per group) as follows; control, free tranilast 200 mg/kg, free tranilast 2 mg/kg, Tranilast/m 4 mg/kg and Tranilast/m 2 mg/kg. 1 % NaHCO_3_ (control) and 200 mg/kg of free tranilast were administered via gavage while 2 mg/kg of free tranilast was given intravenously (i.v.), daily for 6 days. 4 mg/kg of Tranilast/m were administered also intravenously every other day (total 3 cycles) while 2 mg/kg of Tranilast/m were given daily for 6 days.

Antitumor activity of epirubicin in the orthotopic breast cancer models. Preparation and inoculation of 4T1 and E0771 tumor cells was performed as described above. Tumors were allowed to reach a mean volume of 100 mm^3^ and divided into four groups (n=10 per group) as follows: control (NaHCO_3_), Tranilast/m (2 mg/kg), EPI/m (6 mg/kg) and Tranilast/m-EPI/m. Mice were intravenously treated with NaHCO_3_ and Tranilast/m daily for 6 days. Once tumors reached a mean volume of 300 mm^3^, mice received two doses of EPI/m via tail vein injections (i.e., on day 18 and 22 in E0771 tumors, on day 17 and 21 in 4T1 tumors). After tumors of control group have grown to 800 mm^3^, mice were sacrificed, and primary tumors and lungs removed and stored in 1x PBS at -80 ^o^C until further processing.

Antitumor activity of immunotherapy in the orthotopic breast cancer model. Mice bearing E0771 tumors of 100 mm^3^ were randomized in the following groups (n=8-10 per group) and treated with NaHCO_3_/IgG (control group, i.v.), Tranilast/m (2 mg/kg, i.v.), EPI/m (6 or 15 mg/kg, i.v.) or Doxil (3 mg/kg), anti-PD-1 (10 mg/kg)/anti-CTLA-4 (5 mg/kg), Tranilast/m-EPI/m (6 or 15 mg/kg) or Tranilast/m-Doxil (3 mg/kg), Tranilast/m-anti-PD-1 (10 mg/kg)/anti-CTLA-4 (5 mg/kg), anti-PD-1 (10 mg/kg)/anti-CTLA-4 (5 mg/kg)-EPI/m (6 or 15 mg/kg), Tranilast/m-anti-PD-1 (10 mg/kg)/anti-CTLA-4 (5 mg/kg)-EPI/m (6 or 15 mg/kg) or Tranilast/m-anti-PD-1 (10 mg/kg)/anti-CTLA-4 (5 mg/kg)-Doxil (3 mg/kg). Mice received NaHCO_3_ and Tranilast/m tail vain injections for three days (day 11, 12 and 13). EPI/m was administered intravenously while the ICB cocktail and its IgG diluent, intraperitoneally (i.p.) every three days (day 14, 17 and 20) as illustrated in the scheme below. Mice received a second round of Tranilast/m injections on days 15, 16, 18 and 19. A day after completion of treatment protocol, primary tumors of a mean 600 mm^3^ were removed and subjected to further processing.

Mice E0771 rechallenge

Assessment of immunological memory in the Tranilast/m-ICB-EPI/m study: To investigate if mice presenting a complete response after drug treatment have also acquired immunological memory, we rechallenged the animals with orthotopic inoculations of E0771 cancer cells (5x10^4^) 113 days after the initial cancer cell inoculation. As a control group we used 5 mice of the same age to the survivors of Tranilast/m-ICB-EPI/m treatment group. Assessment of immunological memory in the Tranilast/m-ICB-Doxil study: Tumor-free mice from the Tranilast/m-Doxil-ICB (n=8) and Tranilast/m-Doxil (n=3) combination therapy groups were challenged after 90 days from the initial tumor injection with E0771 TNBC cells in the opposite mammary fat pad (left) and on day 130 with MCA205 fibrosarcoma cells (2.5 x10^5^) in the right flank. Naïve C57BL/6 mice of the same age were also injected s.c. with MCA205 or E0771 tumor cells to serve as a control.

Accumulation of tranilast micelles in orthotopic breast cancer primary tumors

BALB/c female mice bearing orthotopic 4T1 tumors of 100 mm^3^ were randomized to two groups (n=5 mice per group) and treated with NaHCO_3_ (control) or 2 mg/kg of Tranilast/m for 5 days. On day 6 of treatment all mice received an i.v. injection of Cy5-labelled Tranilast/m (2 mg/kg), sacrificed and tumors were removed and imaged at 20% excitation power for 5sec at 640 nm excitation and 710 nm emission wavelength using the AMI-HT.

Detection of tranilast in plasma samples

Tranilast/m were injected to 4 BALB/c mice at the tail vein (2 mg/kg on a tranilast basis). At determined time points (0.5, 2, 6, 10 and 24 h), the mice were anesthetized in 3% isoflurane oxygen flow and 50 μl blood sample was collected retro-orbitally and stored in pre-heparinized tubes. Plasma samples were collected after centrifuging the blood at 500g × 15 min. The plasma samples were 5× diluted in DMSO, then filtered through 0.45 μm filter and loaded into an HPLC (solid phase: TSKgel ODS-100V 5 μm; mobile phase: DMF; detector: UV absorbance 340 nm) to detect the tranilast concentration.

Interstitial fluid pressure

Interstitial fluid pressure (IFP) was measured *in vivo* using the previously described wick-in-needle technique after mice were anesthetized with i.p. injection of Avertin and prior to tumor excision ^1^. Briefly, the wick-in-needle equipment consists of a hypodermic needle probe connected to a pressure transducer via a tubing filled with saline. The needle is inserted into the tumor tissue manually. The hole of the needle is filled with nylon threads to improve the fluid communication between the tumor tissue and the probe.

Atomic force microscopy (AFM)

Following completion of the dose response study, E0771 tumors were excised and analyzed with AFM according to the previously described protocol with some modifications^2^. Briefly, samples were harvest and immediately transferred into ice-cold PBS supplemented with a protease inhibitor cocktail (Complete Mini, Roce Dianostics GmbH, 1 tablet per 10 ml). Then, each specimen was immobilized on a 35 mm plastic cell culture petri dish with a thin layer of two-component fast drying epoxy glue. The petri dish was filled with PBS supplemented with the protease inhibitor cocktail and stored at 4 °C to avoid tissue degradation ^3, 4^. AFM measurements were performed with a commercial AFM system (Molecular Imaging-Agilent PicoPlus AFM) between 1-72 h post tumor removal, so as to prevent any alterations in stiffness profiles. The measurements were conducted with silicon nitride cantilevers (MLCT-Bio, cantilever D, Bruker Company). The maximum applied loading force was set to 1.8 nN, the exact spring constant k of the cantilever was determined before each experiment using the thermal tune method and the deflection sensitivity was determined in fluid using petri dishes as an infinitely stiff reference material ^5^. AFM measurements were performed by recording 10-15 different 20 x 20 μm^2^ force maps (16×16 point grids) per specimen, which correspond to 256 force-displacement curves per map with pixel size of 1.25 μm. The collected force maps were analyzed by AtomicJ ^6^ and sample’s Young’s modulus was calculated using the Hertz model.

Ex-vivo elasticity measurements

For the dose response studies of tranilast, elastic modulus of E0771 tumors was measured using an unconfined compression experimental protocol. Following excision of the primary tumor, specimens were loaded on a high precision mechanical testing system (Instron, 5944, Norwood, MA, USA) and compressed to a final strain of 30% with a strain rate of 0.1 mm/min. The dimensions of the primary tumor specimens were 3 × 3 × 2 mm (length × width × thickness) which equal to about one third of the total tumor size. Importantly, tissue from the tumor periphery was excluded to avoid taking measurements corresponding to the collagen capsule that could have led to false conclusions. The elastic modulus was calculated from the slope of the stress-strain curve at the 25-30% strain range ^7^.

In-vivo elasticity measurements

To evaluate *in vivo* the elastic properties of tumors we used shear wave elastography on a Philips EPIQ Elite Ultrasound scanner with an eL18-4 linear array, which is approved for clinical scanning. The method generates a two-dimensional elastic modulus color map after applying a shear wave in the tissue with an acoustic push pulse ^8^. When performing the elastography imaging, a confidence map with highlighted areas of optimal shear wave propagation is produced providing an indication of quality across the stiffness value map. Therefore, we obtained elastic modulus values only from the region of interest (ROI) with the highest shear wave quality. The elastic modulus value presented in the results is the average value of ROI. For the EPI/m studies, shear wave imaging was performed, on day 11, 14, 18 and 24 post-treatment for E0771 and on day 11, 14, 17 and 24 for 4T1 model. The ultrasound imaging of E0771 tumors for the Tranilast/m-EPI/m (or Doxil)-ICB study was performed prior to epirubicin micelles administration, on day 14 and 21 post-epirubicin treatment.

Fluorescent immunohistochemistry

Tumor samples were washed twice in 1x PBS for 10 min and incubated with 4 % PFA overnight at 4 °C. The fixative was aspirated, and samples washed twice in 1x PBS for 10 min. Fixed tissues were embedded in optimal cutting temperature compound in cryomolds (Tissue-Tek) and frozen completely at -20 °C. Transverse 30 μm thick tumor sections were produced using the Tissue-Tek Cryo3 (SAKURA). Positively charged HistoBond microscope slides (Marienfeld) were used to bond four tissue sections per tumor.

For collagen I and hyaluronan detection, tumor sections were incubated in blocking solution (10 % fetal bovine serum, 3 % donkey serum, 1× PBS) for 2 h and then immunostained with rabbit anti-Collagen I (ab4710, Abcam, 1:100) and sheep anti-Hyaluronan (ab53842, Abcam, 1:100) primary antibodies overnight at 4 °C. The next day, slides were washed in 1x PBS and incubated with Alexa Fluor-647 anti-rabbit IgG (H+L) (A21244, Invitrogen, 1:400) and anti-sheep Alexa Fluor-488 IgG (H+L) (A11015, Invitrogen, 1:400) secondary antibodies and DAPI stain (Sigma, 1:100 of 1 mg/ml stock) for 2 h at room temperature (RT) in the dark. Sections were mounted on microscope slides using the ProLong gold antifade mountant (Invitrogen) and covered with a glass coverslip.

Functional vasculature of E0771 primary tumors was assessed following anesthetization of mice with i.p. injection of Avertin (200 mg/kg) and intracardial injection of 100 μl biotinylated lycopersicon esculentum lectin (4 mg/kg, B-1175, Vector Labs) which was allowed to distribute throughout the body for 7 min. Then, mice were sacrificed via CO_2_ inhalation and excised tumors were fixed and processed as described above for IHC analysis. The number of blood vessels was measured from the positive staining of the endothelial marker CD31 (MEC13.3, BD Pharmingen, 1:100) while the fraction of perfused vessels was determined as the ratio of lectin and CD31 overlapping staining to CD31 positive staining. CD31 signal was detected with Alexa Fluor-647 goat anti-rat IgG (H+L) (A21247, Invitrogen, 1:400) secondary antibody and lectin signal with Streptavidin Alexa Fluor 488 conjugate (S11223, Invitrogen, 1:1000).

For CAF assessment, E0771 tumors were fixed, dehydrated in successive alcohol steps and xylene. Then, tumors were embedded in paraffin. Paraffin tissue sections of 7 μm were produced using the microtome (Accu-Cut SRM 200 Rotary Microtome, SAKURA), flatten out into water and allowed to dry overnight at 37 °C. Sections were then deparaffinized and rehydrated as routinely processed for histology. CAF proliferation was evaluated by immunostaining for the presence of αSMA (ab5694, Abcam, 1:50) protein and biotinylated Ki67 (13-5698-82, Invitrogen, 1:100) after antigen retrieval and blocking (10% FBS, 3% donkey serum in 1x PBS). After overnight incubation at 4 with primary antibodies, tissue sections were washed in TBS-T and incubated for 2 h at RT with donkey anti-rabbit Alexa Fluor 555 (**A31572,** Invitrogen, 1:400) and Streptavidin Alexa Fluor 488 conjugate (S11223, Invitrogen, 1:1000) and DAPI stain. Tissue sections were then mounted on microscope slides and pictures were taken using the STELLARIS 5 confocal microscope. One tumor section was assessed per mouse and 22-30 image fields were captured per tissue section. The mean signal intensity of total image fields produced from a single tissue section was used for quantification.

Histological image acquisition

Images of stained tumor sections from the tumor interior and periphery were acquired at 10x magnification using an Olympus BX53 fluorescence microscope, unless otherwise stated. To enable quantification, images of the same staining were taken at identical settings. The images were analyzed using custom and built-in algorithms in MATLAB (MathWorks, Inc., Natick, MA, USA).

Lung metastasis quantification

Following completion of treatment protocol #2, lungs were excised and fixed in Bouin’s solution for 72 h and lung surface metastases were identified by a light-yellow color. All visible surface metastases were quantified by masked counting.

Hematoxylin and eosin

Lungs were excised from mice, which were rechallenged with E0771 cells, at the study endpoint and fixed in 4% PFA, dehydrated through a series of graded ethanol washes and embedded in paraffin. Transverse 7 μm-thick tissue sections were produced using the microtome (Accu-Cut SRM 200 Rotary Microtome, SAKURA), flatten out into water and allowed to dry overnight at 37 °C. Sections were then deparaffinized, rehydrated and stained with Harris hematoxylin (Sigma) for 10 min and then incubated with eosin (Sigma) until they become pink.

Flow cytometry

On day 21 of treatment E0771 breast tumors (n=5-10 per treatment group) were harvested in 1x PBS, minced to fine fragments and incubated with Accumax (Millipore) for 1 h at RT on an end-over-end shaker. Enzymatic digestion was ceased by the addition of RPMI media containing 10% FBS and 1% antibiotic /antimycotic solution. The resulting tissue homogenates were filtered through 40 μm cell strainers and single cell suspensions were collected and counted. Cell suspensions were then incubated with fixable viability dye-e780 (Invitrogen, 65-0865-14, 1:3000) for gating of viable cells. Non-specific antibody binding was blocked following incubation with the rat anti-mouse CD16/CD32 mAb (BD Bioscience, ﻿553142, 12.4G2, 10 μg/ml) for 10min at room temperature. 1x10^6^ cells per sample were labeled with the various fluorochrome conjugated antibodies, washed and resuspended in 1% BSA, 1x PBS buffer. The anti-mouse antibodies used in the experiment are the following; CD4-AF700 (BioLegend, 100429, GK1.5, 0.312 μg/ml), CD127-APC (BioLegend, 135011, A7R34, 2.5 μg/ml), IgG2a-APC (BioLegend, 400511, RTK2758), CD8a-e450 (eBioscience, 48-0081-80, 53-6.7, 1.25 μg/ml), Foxp3-PE (BD Bioscience, 560414, MF23, 1 μg/test), CD45-V500 (﻿BD Bioscience, 561487, 30-F11, 1.25 μg/ml), CD25-PE-Cy7 (BD Bioscience, 552880, PC61, 5 μg/ml), CD3-PE/Dazzle 594 (BD Bioscience, 100347, 145-2C1, 5 μg/ml), CD11b-e450 (eBioscience, 48-0112-82, M1/70, 0.312 μg/ml), Gr-1-PE (BioLegend, 108407, RB6-8C5, 0.625 μg/ml), F4/80-APC (BioLegend, 123116, BM8, 2.5 μg/ml), CD206-PE-Cy7 (BioLegend, 141720, C068C2, 5 μg/ml), MHCII-FITC (BioLegend, 107605, M5/114.15.2, 0.625 μg/ml). Flow cytometry data were obtained using BD FACSAria™ III flow cytometer and analysed using BD FACS Suite software. Data presented are representative of singlets, live cells.

**Supplementary Figures**


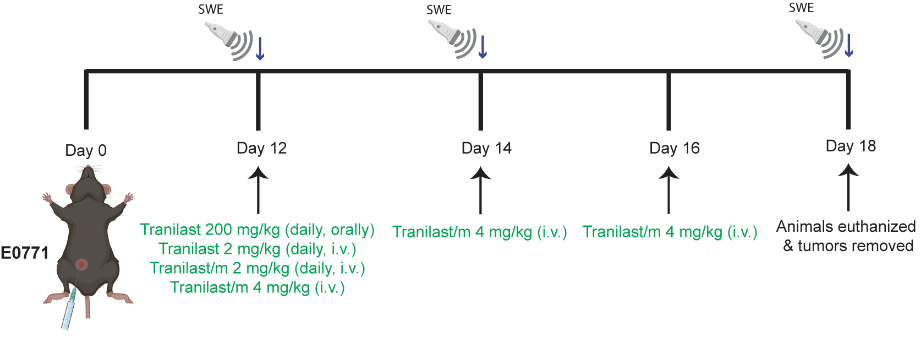


**Supplementary Figure 1.** Treatment protocol #1. Created with [BioRender.com](http://BioRender.com).


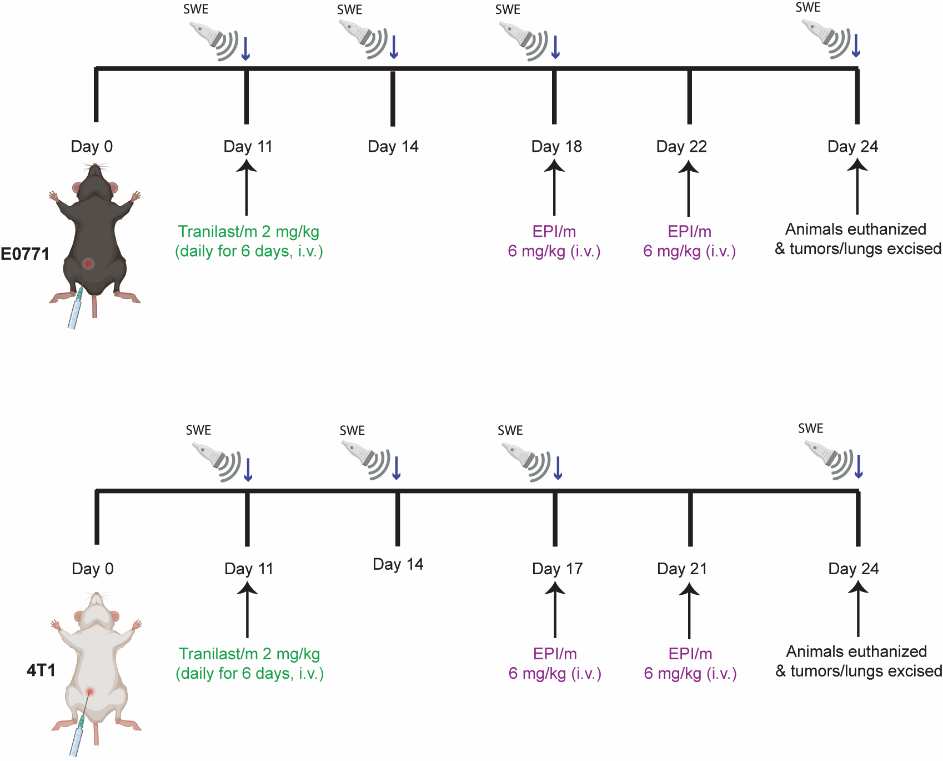


**Supplementary Figure 2.** Treatment protocol #2. Created with [BioRender.com](http://BioRender.com).


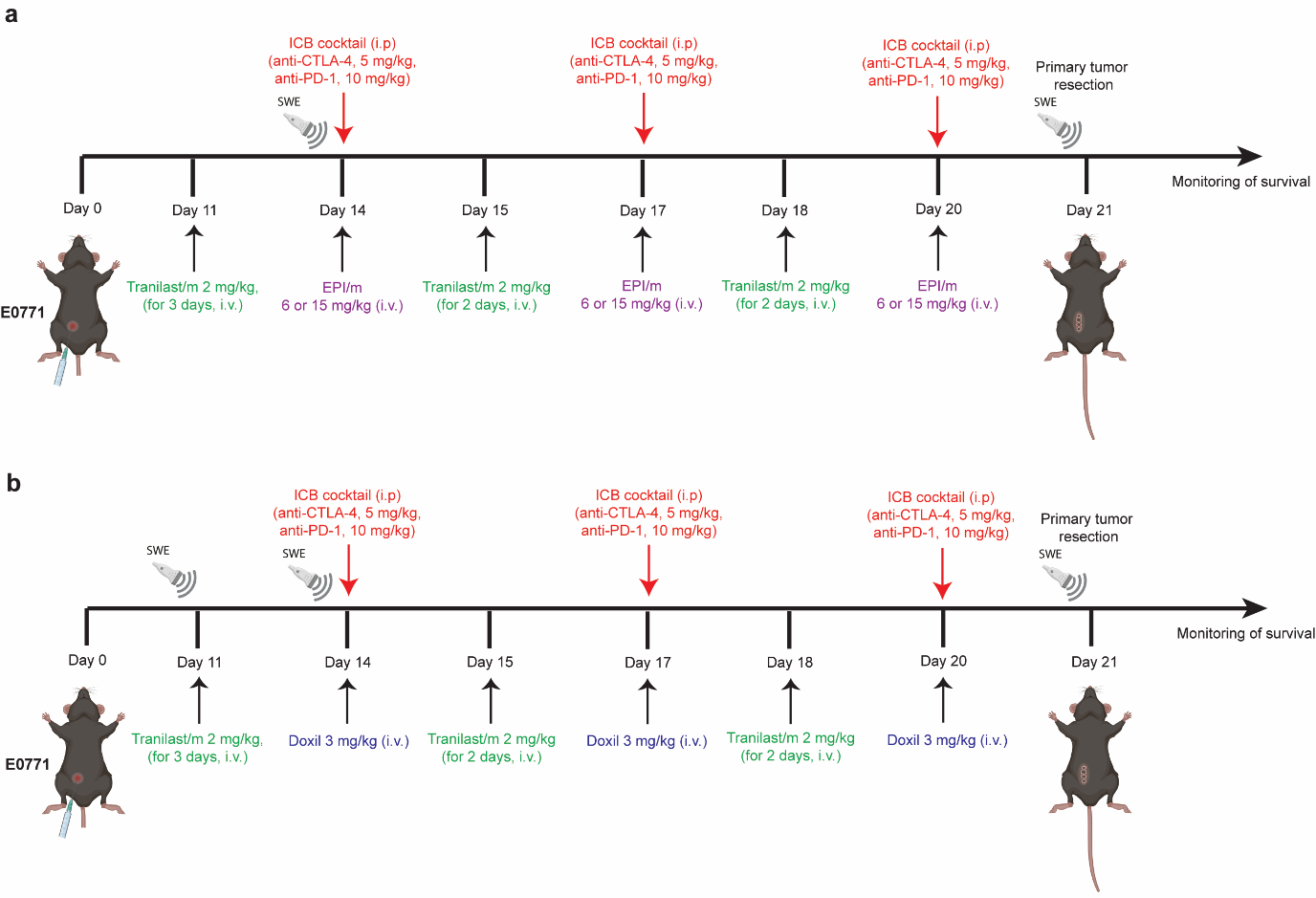


**Supplementary Figure 3.** Treatment protocol #3 (**a**) for EPI/m study and (**b**) for Doxil study. Created with [BioRender.com](http://BioRender.com).


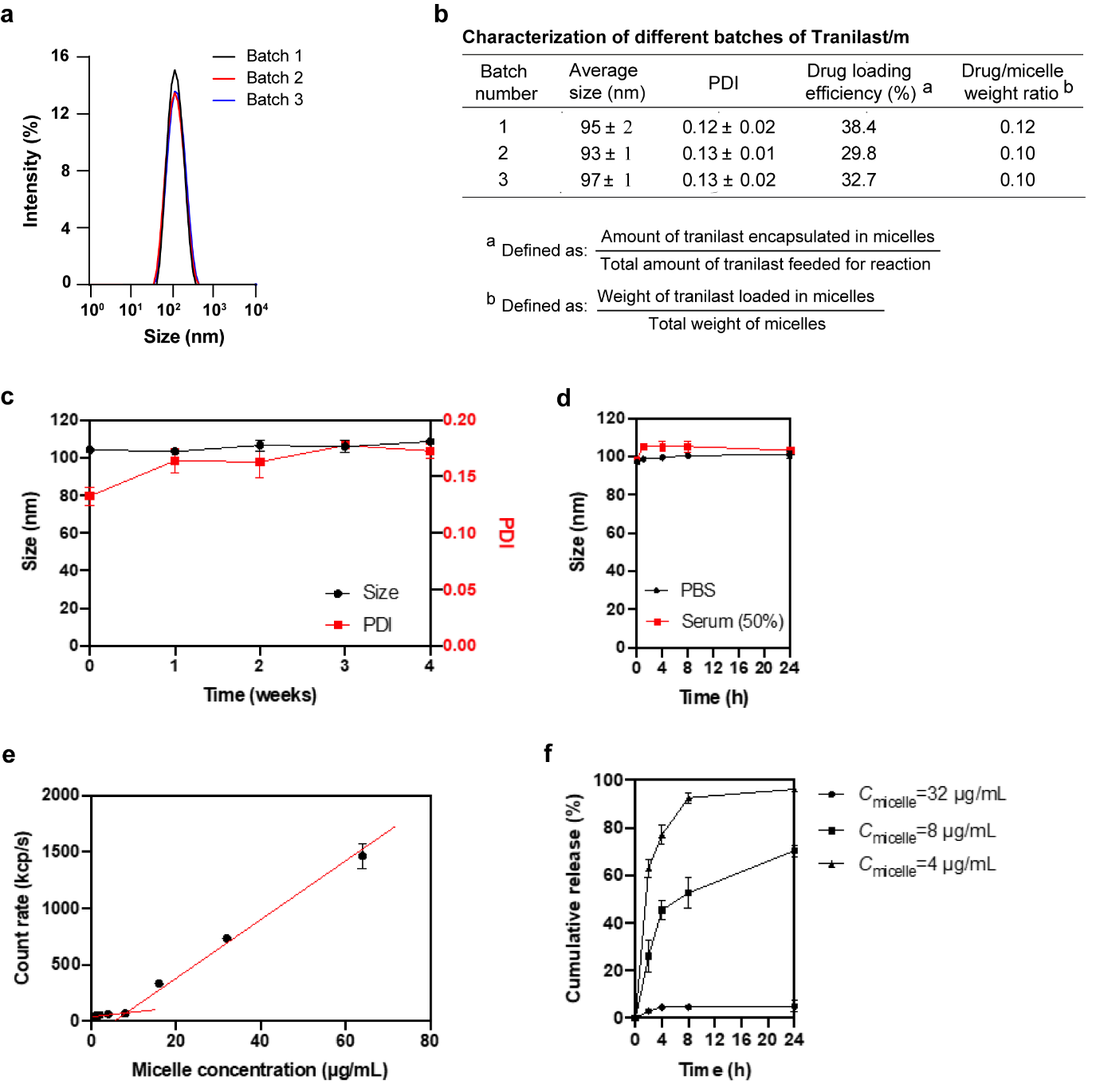


**Supplementary Figure 4.** Tranilast micelles *in vitro* characterization. (**a**) Representative dynamic light scattering (DLS) results of Tranilast/m prepared from three different batches of reaction. The samples shared comparable size distribution. (**b**) Characterization of different batches of Tranilast/m.

(**c**) DLS measurement results indicating the stability of Tranilast/m upon long time storage. The Tranilast/m were incubated in pure water and stored at 4℃. Data shown as the mean ± SD (n=3). (**d**) Time-dependent change of the average size of Tranilast/m after dilution to a lower concentration (0.1 mg/ml on micelle basis) in PBS and 50% serum. Data shown as the mean ± SD (n=3 independent experiments). (**e**) Critical micelle concentration (CMC) as defined by scattering light intensity. Data shown as the mean ± SD (n=3 independent experiments). (**f**) Drug release profile of Tranilast/m at different micelle concentrations in PBS (pH 7.4) containing 20% serum at 37 ℃. Data shown as the mean ± SD (n=3 independent experiments).

**
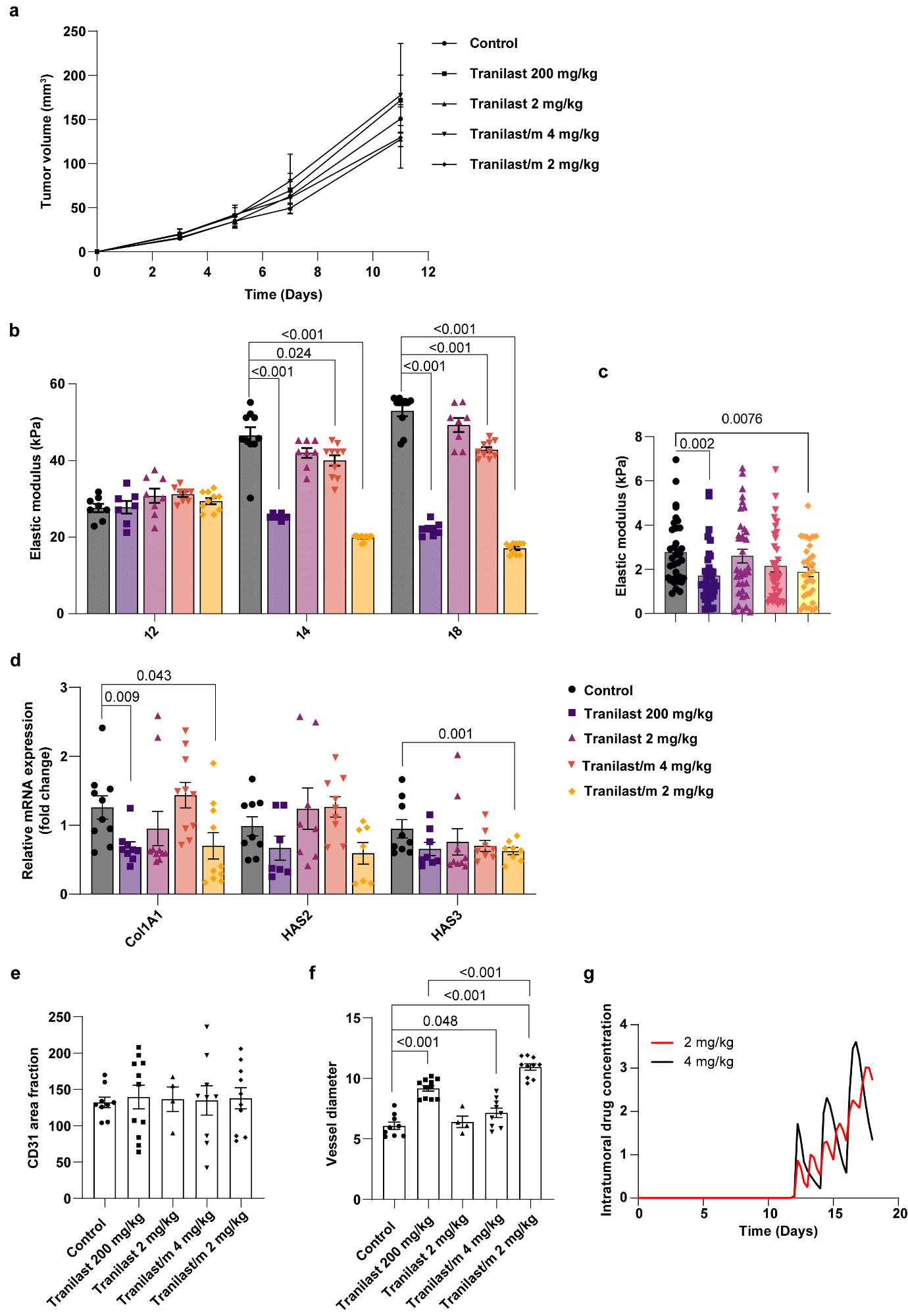
**

**Supplementary Figure 5.** Tranilast micelles treatment reduces tumor stiffness and increases vessel diameter without interfering with tumor growth and angiogenesis. (**a**) Orthotopic E0771 tumor growth over an 11-day period following different treatments (n=4 mice). (**b**) Elastic modulus values in E0771 tumors before (day 12), during (day 14) and upon completion of the 6-day treatment (day 18) using ultrasound shear wave elastography (n=8-10 mice, N=2 image fields per mouse). (**c**) Assessment of nanoscale changes in elastic modulus of E0771 tumors upon completion of treatment by Atomic Force Microscopy. (n=3 samples per treatment group, N=12 areas/regions of interests-ROI (20x20 μm) were characterized. Thus, for each group you will find ~36 measurements (3 samples x 12 ROI). (**d**) Relative mRNA expression levels of *Col1A1*, and *hyaluronan synthase 2* (*HAS2*) and *3* (*HAS3*) in E0771 tumors assessed by RT-qPCR (n=3 mice, N=2-3 technical replicates) following different treatments. Data presented as mean ± SE. Statistical analyses were performed by comparing means between two independent groups using the unpaired parametric Welch t-test. (**e**) Quantification of CD31 area fraction following immunostaining of E0771 tumor samples with anti-CD31 endothelial cell marker. (**f**) Quantification of vessel diameter of E0771 tumors treated as indicated. Data are presented as mean ± SE. Statistical analyses were performed by comparing means between two independent groups using the unpaired parametric Welch t-test. (**g**) Mathematical model predictions for the temporal variations of the average intratumoral concentration (in dimensionless units) of tranilast administered via the 2 mg/kg (daily) and the 4mg/kg (every second day) dose schedule of Tranilast/m as shown in Supplementary Fig. 1.


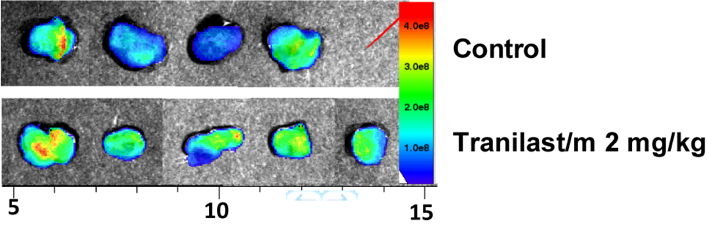


**Supplementary Figure 6.** Tumor tissue distribution of the micelles in control and Tranilast/m treated mice 24 h after injection of Cy5-micelles (n=4-5 mice).


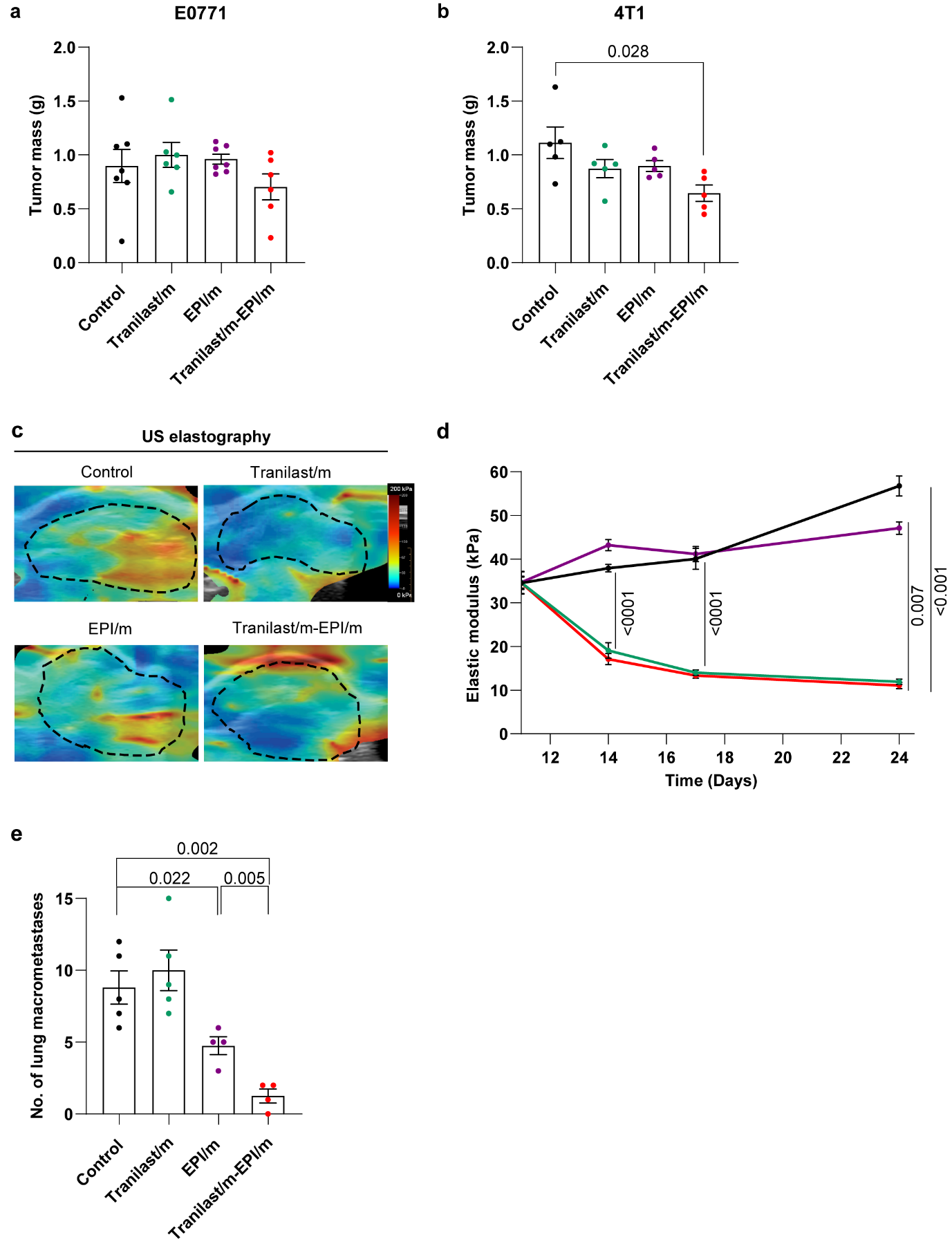


**Supplementary Figure 7.** Tranilast micelles enhance the efficacy of epirubicin micelles and decrease lung metastasis. Orthotopic E0771 (**a**) and 4T1 (**b**) breast cancer primary tumor mass in mice treated with Tranilast/m 2 mg/kg, EPI/m 6 mg/kg or their combination. Animals received daily i.v. injections of Tranilast/m for 6 days and two doses of EPI/m (on days 18 and 22 in E0771 tumor model and on days 17 and 21 in 4T1 tumor model). (**c**) Representative ultrasound elastography heat maps of 4T1 tumors following different treatments, with blue indicating compliant tissue and red indicating stiff tissue. The dashed black line denotes the tumor margin (n=4-5, N=2 image fields per mouse). (**d**) Elastic modulus values in 4T1 tumors using ultrasound elastography. (**e**) Quantification of macrometastases in the lungs of 4T1 tumor model (n=5 mice). Data are presented as mean ± SE. Statistical analyses were performed by comparing means between two independent groups using the unpaired parametric Welch t-test.


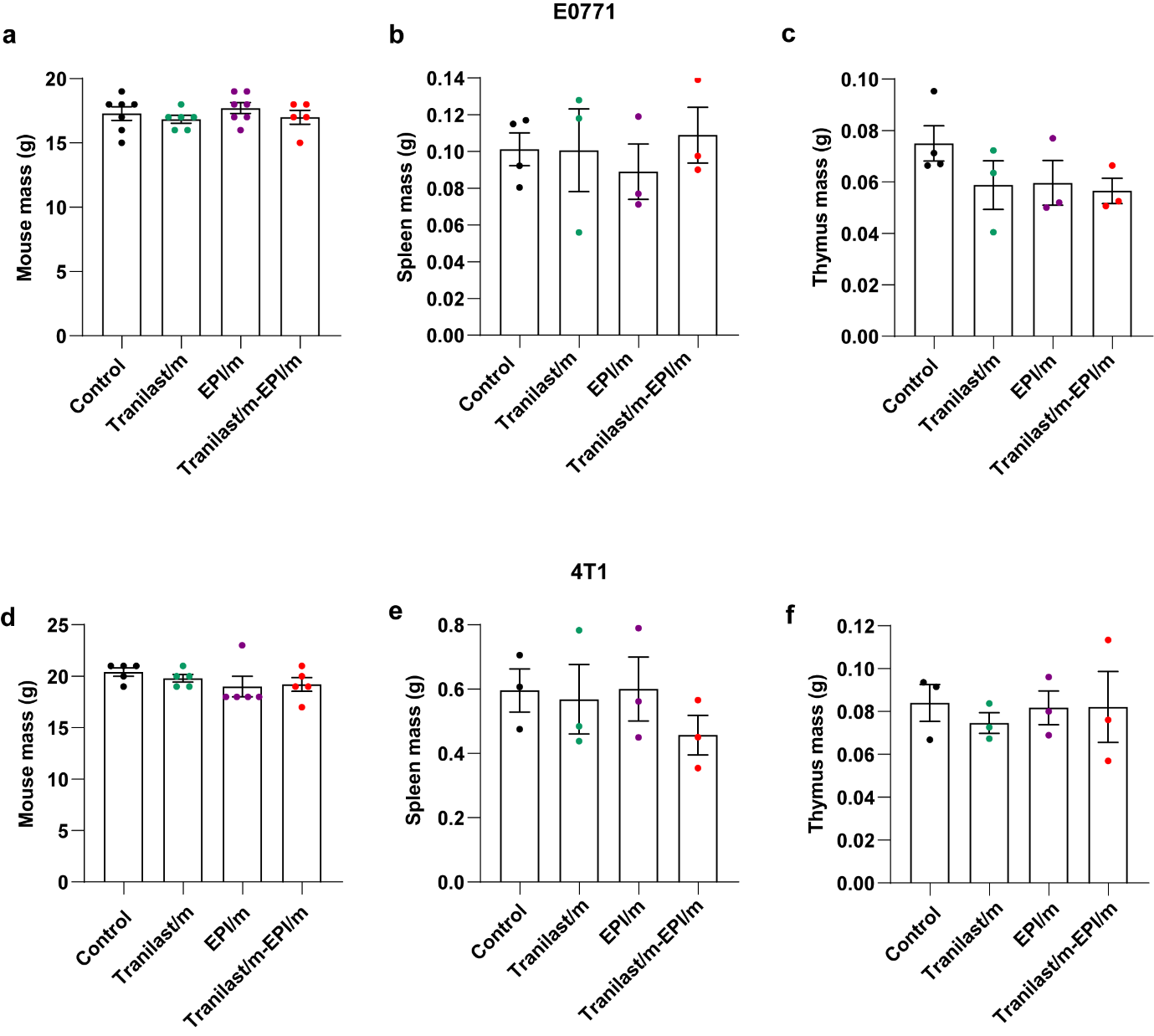


**Supplementary Figure 8.** Mouse, spleen and thymus mass measurements in E0771 and 4T1 tumor bearing mice. No significant body weight loss (**a**, **d**), spleen mass (**b**, **e**) and thymus mass (**c**, **f**) were detected in any of the mice at study endpoint (n=3-5 mice). Data are presented as mean ± SE. Statistical analyses were performed by comparing means between two independent groups using the unpaired parametric Welch t-test.

**
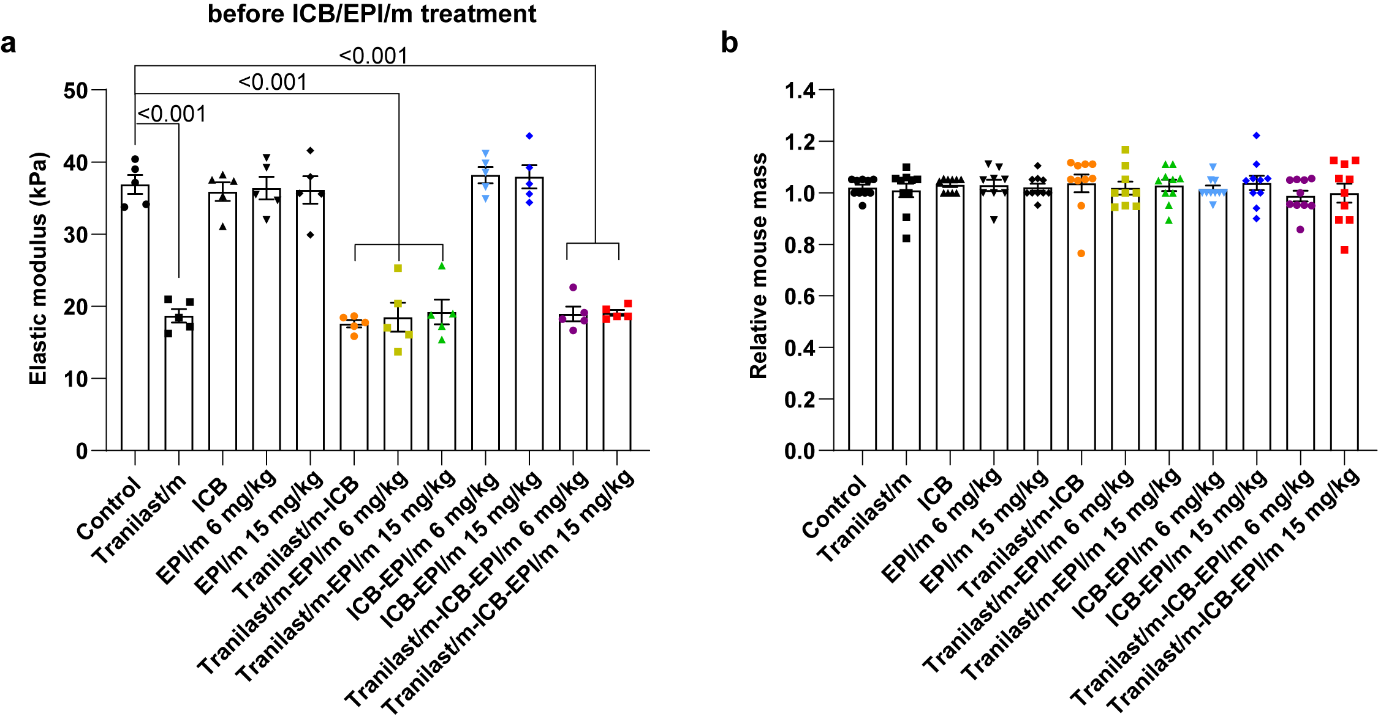
**

**Supplementary Figure 9.** (**a**) Tumor elastic modulus in E0771 tumors on day 14, prior to chemotherapy and immunotherapy treatment (n=5 mice, N=2 image fields per mouse). (**b**) Mouse mass measured at the completion of the treatment protocol normalized to the mass of the control mice. No significant animal weight loss was detected (n=9-10 mice). Data are presented as mean ± SE. Statistical analyses were performed by comparing means between two independent groups using the unpaired parametric Welch t-test.


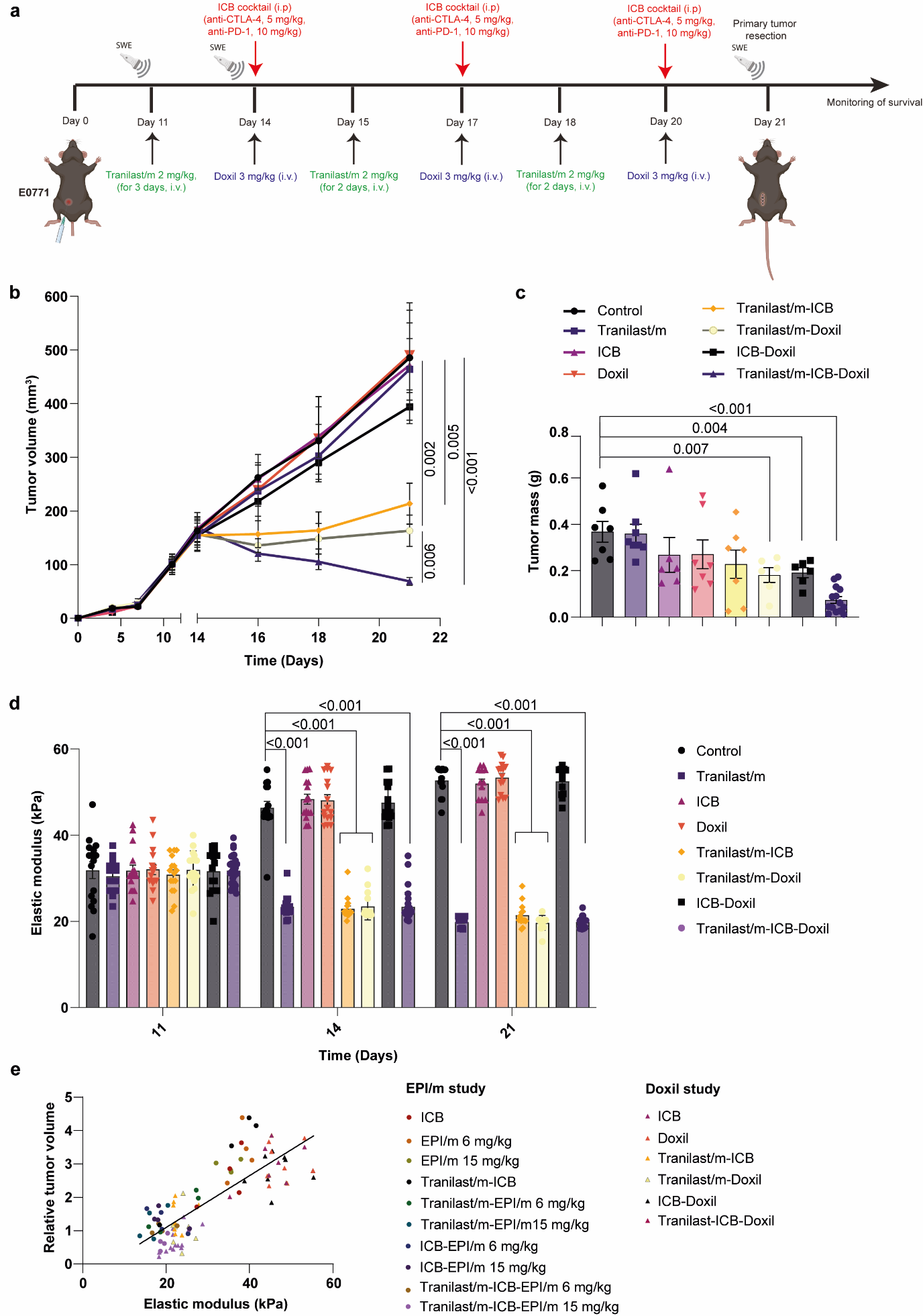


**Supplementary Figure 10.** (**a**) Study treatment protocol for the effect of tranilast micelles on nano-immunotherapy. (**b**) Tumor volume and (**c**) mass following excision of E0771 tumors treated as indicated. Tranilast treatment potentiates the antitumor activity of Doxil and ICB (n=8 mice). (**d**) Tumor elastic modulus in E0771 tumors prior to any treatment (day 11), after three days of Tranilast/m and before ICB or Doxil treatment (day 14) and following three doses of nano- and immunotherapy (day 21). Tranilast/m were daily administered for three days and between doses of Doxil and ICB which were given every three days (n=16 mice). Data are presented as mean ± SE. For **b-d**, statistical analyses were performed by comparing means between two independent groups using the unpaired parametric Welch t-test. (**e**) Relative change in primary tumor volume at the end of the experiment compared to tumor volume prior to initiation of the treatments (EPI/m, Doxil) as a function of the elastic modulus before treatment initiation (R^2^=0.66, quantifiable analysis of how well the line of best fit, fits the data). Supplementary Fig 10a was created with [BioRender.com](http://BioRender.com).


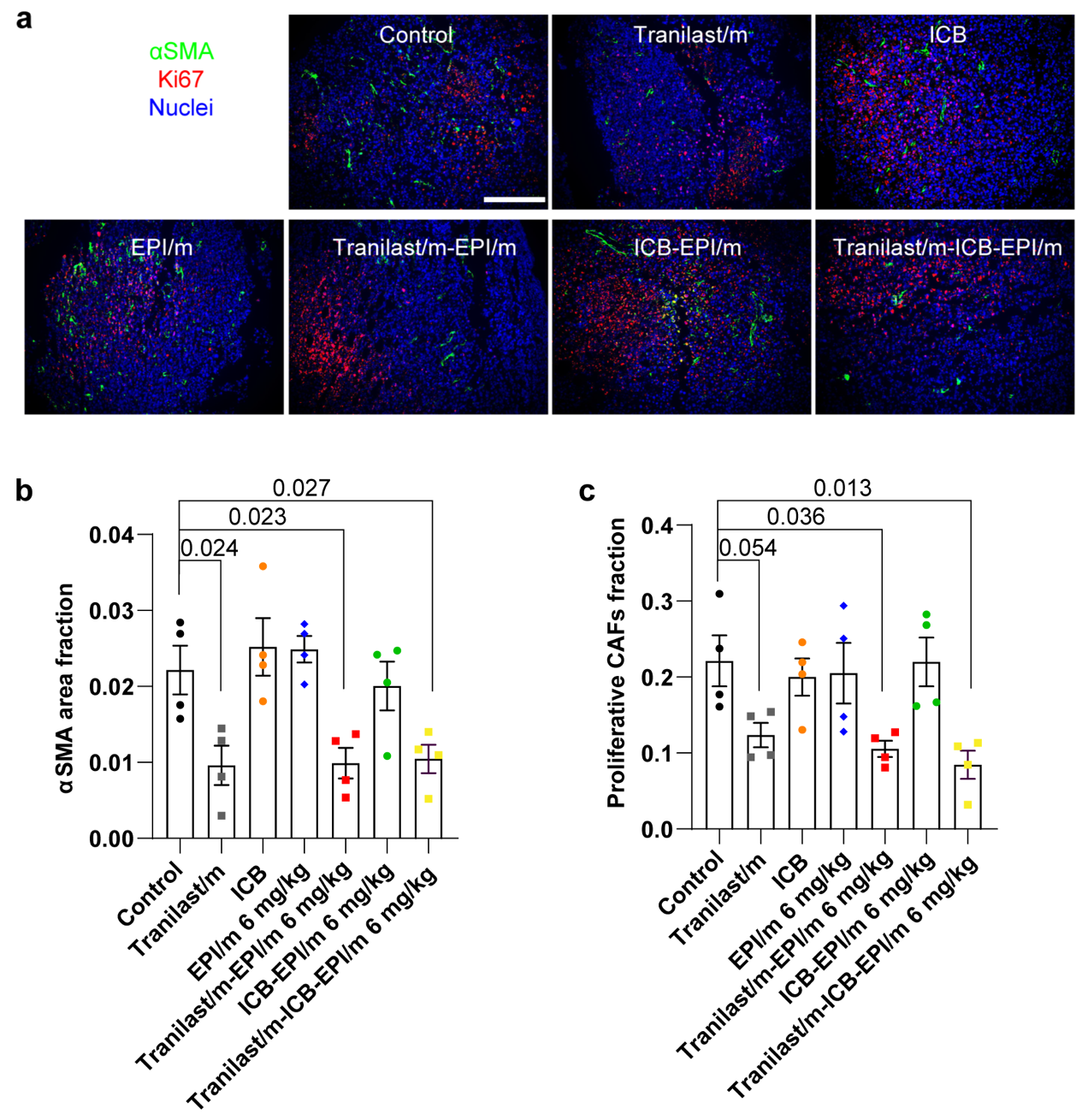


**Supplementary Figure 11.** (**a**) Immunofluorescence staining of E0771 tumor tissues using the CAF marker anti-αSMA (green) and proliferation marker anti-Ki67 (red). (**b**) Quantification of αSMA positive fraction normalised to DAPI nuclear stain. (**c**) Quantification of area double positive for αSMA and Ki67 marker as a measure of CAF proliferation normalized to total αSMA positive staining. For the image acquisition the confocal microscope Leica STELLARIS was used to scan the entire tissue section (corresponding to total 22-30 image fields) per mouse. Data are presented as mean ± SE. Statistical analyses were performed by comparing means between two independent groups using the unpaired parametric Welch t-test (n=4 mice, scale bar 200 nm).


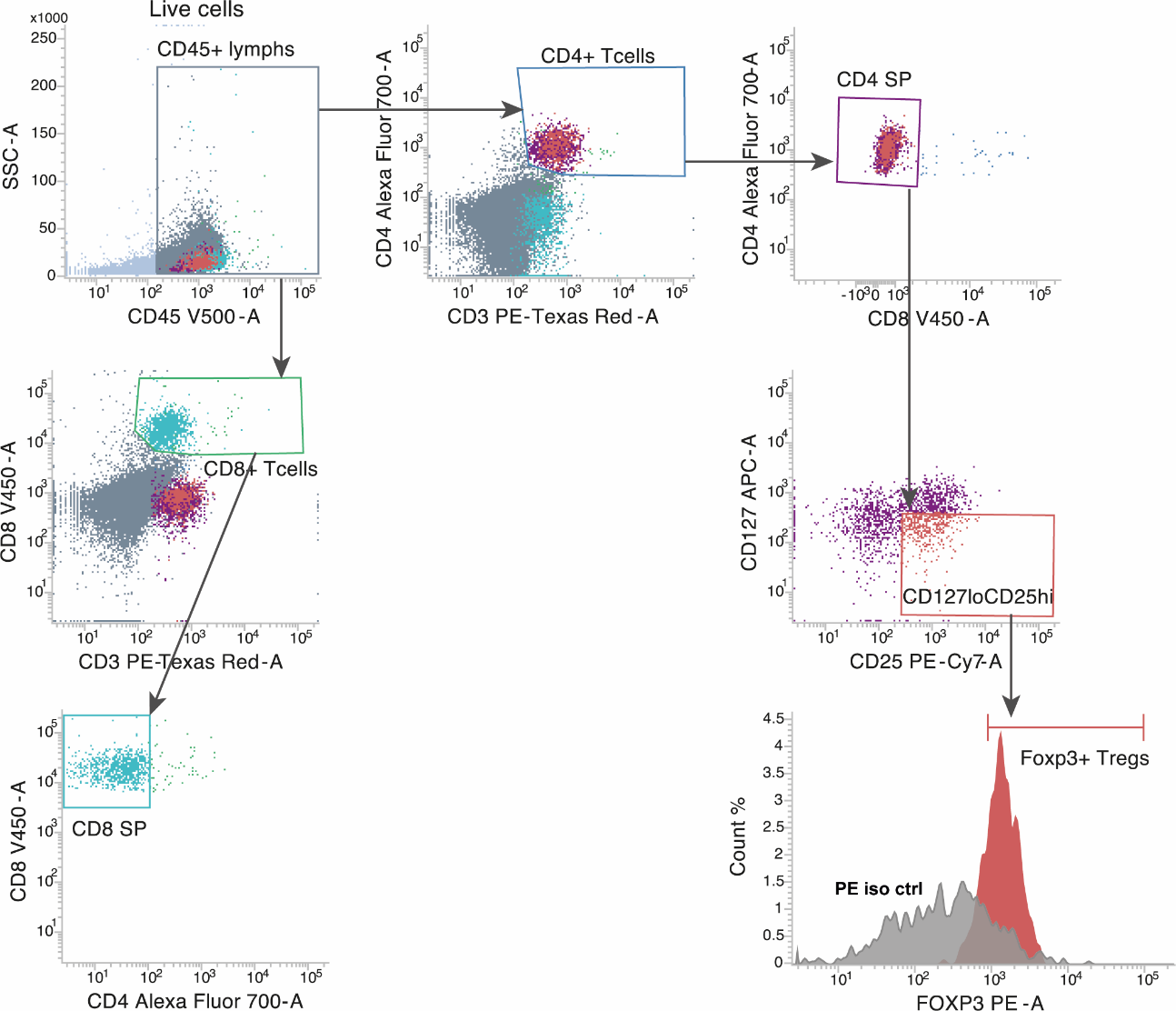


**Supplementary Figure 12.** Flow cytometry gating strategy for T cell immunophenotyping in E0771 tumors.

**Supplementary Figure 13.** Lungs of survived E0771 bearing mice with no evidence of macroscopic metastases. Lungs of 3 of the survivors of the Tranilast/m-ICB-EPI/m 15 mg/kg treatment group were collected to study the presence of metastasis.

**Supplementary References**

1. Boucher, Y., Baxter, L. T. & Jain, R. K. Interstitial pressure gradients in tissue-isolated and subcutaneous tumors: implications for therapy. *Cancer Res.* **50**, 4478-4484 (1990).

2. Stylianou, A., Lekka, M. & Stylianopoulos, T. AFM assessing of nanomechanical fingerprints for cancer early diagnosis and classification: from single cell to tissue level. *Nanoscale* **10**, 20930-20945 (2018).

3. Tian, M. *et al*. The nanomechanical signature of liver cancer tissues and its molecular origin. *Nanoscale* **7**, 12998-13010 (2015).

4. Plodinec, M. *et al*. The nanomechanical signature of breast cancer. *Nature nanotechnology* **7**, 757-765 (2012).

5. Stylianou, A., Gkretsi, V., Patrickios, C. S. & Stylianopoulos, T. Exploring the Nano-Surface of Collagenous and Other Fibrotic Tissues with AFM. *Fibrosis* **1627***,* 453-489 (Springer, 2017).

6. Hermanowicz, P., Sarna, M., Burda, K. & Gabryś, H. AtomicJ: an open source software for analysis of force curves. *Rev. Sci. Instrum.* **85**, 063703 (2014).

7. Voutouri, C. & Stylianopoulos, T. Accumulation of mechanical forces in tumors is related to hyaluronan content and tissue stiffness. *PloS one* **13**, e0193801 (2018).

8. Sigrist, R. M., Liau, J., El Kaffas, A., Chammas, M. C. & Willmann, J. K. Ultrasound elastography: review of techniques and clinical applications. *Theranostics* **7**, 1303 (2017).
