# Supplementary material for: Polymeric micelles effectively reprogram the tumor microenvironment to potentiate nano-immunotherapy in mouse breast cancer models
Source: Nat Commun. 2022 Nov 22;13:7165. doi: 10.1038/s41467-022-34744-1 (PMC9684407; doi:10.1038/s41467-022-34744-1)
Supplement: Supplementary file 2 — Reporting Summary [file 41467_2022_34744_MOESM2_ESM.pdf]

nature portfolio

Corresponding author(s): Triantafyllos Stylianopoulos and Horacio Cabral

Last updated by author(s): Sep 22, 2022

Reporting Summary

Nature Portfolio wishes to improve the reproducibility of the work that we publish. This form provides structure for consistency and transparency in reporting. For further information on Nature Portfolio policies, see our Editorial Policies and the Editorial Policy Checklist.

Statistics

For all statistical analyses, confirm that the following items are present in the figure legend, table legend, main text, or Methods section.

n/a	Confirmed	
☐	☒	The exact sample size (*n*) for each experimental group/condition, given as a discrete number and unit of measurement
☐	☒	A statement on whether measurements were taken from distinct samples or whether the same sample was measured repeatedly
☐	☒	The statistical test(s) used AND whether they are one- or two-sided<br>*Only common tests should be described solely by name; describe more complex techniques in the Methods section.*
☒	☐	A description of all covariates tested
☐	☒	A description of any assumptions or corrections, such as tests of normality and adjustment for multiple comparisons
☐	☒	A full description of the statistical parameters including central tendency (e.g. means) or other basic estimates (e.g. regression coefficient) AND variation (e.g. standard deviation) or associated estimates of uncertainty (e.g. confidence intervals)
☐	☒	For null hypothesis testing, the test statistic (e.g. *F*, *t*, *r*) with confidence intervals, effect sizes, degrees of freedom and *P* value noted<br>*Give P values as exact values whenever suitable.*
☒	☐	For Bayesian analysis, information on the choice of priors and Markov chain Monte Carlo settings
☒	☐	For hierarchical and complex designs, identification of the appropriate level for tests and full reporting of outcomes
☒	☐	Estimates of effect sizes (e.g. Cohen's *d*, Pearson's *r*), indicating how they were calculated

Our web collection on statistics for biologists contains articles on many of the points above.

Software and code

Policy information about availability of computer code

Data collection	BD FACSAria III (BD Biosciences), QLab software (Phillips Medical Systems) , AMI HT aura software (Spectral Instruments Imaging)
Data analysis	GraphPad Prism 8 (GraphPad Software Inc.), MATLAB (MathWorks, Inc.), BD FACSUITE software (BD Biosciences), Microsoft Excel (Microsoft)

For manuscripts utilizing custom algorithms or software that are central to the research but not yet described in published literature, software must be made available to editors and reviewers. We strongly encourage code deposition in a community repository (e.g. GitHub). See the Nature Portfolio guidelines for submitting code & software for further information.

Data

Policy information about availability of data

All manuscripts must include a data availability statement. This statement should provide the following information, where applicable:

- Accession codes, unique identifiers, or web links for publicly available datasets
- A description of any restrictions on data availability
- For clinical datasets or third party data, please ensure that the statement adheres to our policy

Al source data are provided with this paper.

Human research participants

Policy information about studies involving human research participants and Sex and Gender in Research.

Reporting on sex and gender	*Use the terms sex (biological attribute) and gender (shaped by social and cultural circumstances) carefully in order to avoid confusing both terms. Indicate if findings apply to only one sex or gender; describe whether sex and gender were considered in study design whether sex and/or gender was determined based on self-reporting or assigned and methods used. Provide in the source data disaggregated sex and gender data where this information has been collected, and consent has been obtained for sharing of individual-level data; provide overall numbers in this Reporting Summary. Please state if this information has not been collected. Report sex- and gender-based analyses where performed, justify reasons for lack of sex- and gender-based analysis.*
Population characteristics	*Describe the covariate-relevant population characteristics of the human research participants (e.g. age, genotypic information, past and current diagnosis and treatment categories). If you filled out the behavioural & social sciences study design questions and have nothing to add here, write "See above."*
Recruitment	CAFs were obtained from surgically resected lung adenocarcinoma patients at National Cancer Center Hospital East.
Ethics oversight	Experiments using human materials were approved by National Cancer Center Institutional Review Board (2005-043).

Note that full information on the approval of the study protocol must also be provided in the manuscript.

Field-specific reporting

Please select the one below that is the best fit for your research. If you are not sure, read the appropriate sections before making your selection.

☒ Life sciences ☐ Behavioural & social sciences ☐ Ecological, evolutionary & environmental sciences

For a reference copy of the document with all sections, see nature.com/documents/nr-reporting-summary-flat.pdf

Life sciences study design

All studies must disclose on these points even when the disclosure is negative.

Sample size	No statistical method was used to predeterminesample sizes. n=3 was chosen as minimal number of biologically independent groups per experiment for the in vitro studies.<br>For animal studies, sample size was chosen based on our previous research (M. Panagi et al, Theranostics 2020 and F. Mpekris et al., Advanced Science 2020). GraphPad PRISM software was used for statistical analyses. We observed many statistically significant effects in the data (using unpaired t test, p<0.05), indicating that the effective sample size was sufficient for studying the phenomena of interest.<br>Individual data points on graphs represent individual biological replicates.
Data exclusions	No data were excuded form analysis.
Replication	All in vitro experiments were replicated at least three time with fresh biologcal replicates.<br>For in vivo studies, two murine models of TNBC: 4T1 and E0771 were used.<br>All attempts at replication were successful.
Randomization	Allocation of cells in vitro experimental procedures was done at random. For in vivo studies, size-matched tumor bearing mice were randomly assigned to treatment groups. For histopathological and microscopical examinations samples were blinded with alpha-numeric codes to ensure unbiased imaging and analyses.
Blinding	Although tumor growth measurements were carried out by a single investigator to reduce the impact of human error, all histology and microscopy examinations were performed by different investigators and were blinded with alpha-numeric codes to ensure unbiased imaging and analysis.

Reporting for specific materials, systems and methods

We require information from authors about some types of materials, experimental systems and methods used in many studies. Here, indicate whether each material, system or method listed is relevant to your study. If you are not sure if a list item applies to your research, read the appropriate section before selecting a response.

Materials & experimental systems

n/a	Involved in the study
☐	☒ Antibodies
☐	☒ Eukaryotic cell lines
☒	☐ Palaeontology and archaeology
☐	☒ Animals and other organisms
☒	☐ Clinical data
☒	☐ Dual use research of concern

Methods

n/a	Involved in the study
☐	☐ ChIP-seq
☐	☒ Flow cytometry
☐	☐ MRI-based neuroimaging

Antibodies

Antibodies used

anti-Collagen I (Abcam, ab34710, lot: GR3241980-1)
anti-Hyaluronan (Abcam, ab53842, lot: GR323866-7)
anti-CD31 (BD Pharmingen, 553370, MEC13.3., lot: 4101830)
anti-αSMA (Abcam, ab5694, lot: GR3356867-9)
anti-Ki67 (Invitrogen, 13-5698-82, lot:2373799)

Alexa Fluor-647 goat anti-rabbit IgG (H+L) (Invitrogen, A21244, lot: 1620162)
Alexa Fluor-488 donkey anti-sheep IgG (H+L) (Invitrogen, A11015, lot: 2155293)
Alexa Fluor-647 goat anti-rat IgG (H+L) (Invitrogen, A21247, lot: 1524904)
Alexa Fluor-555 donkey anti-rabbit IgG (H+L) (Invitrogen, A31572)
Streptavidin Alexa Fluor-488 conjugate (Invitrogen, S11223, lot:1922572)
DAPI (Sigma Aldrich, 10236276001, lot:48001422)

FVD-e780 (Invitrogen, 65-0865-14, lot:2185428)
CD16/CD32 (BD Bioscience, 553142, 12.4G2, lot:0148675)
CD4-AF700 (BioLegend, 100429, GK1.5, lot:B288422)
CD127-APC (BioLegend, 135011, A7R34, lot:B313098)
IgG2a-APC (BioLegend, 400511, RTK2758, lot: B259641)
CD8a-e450 (eBioscience, 48-0081-80, 53-6.7, lot:1998363)
Foxp3-PE (BD Bioscience, 560414, MF23, lot: 9344549)
CD45-V500 (BD Bioscience, 561487, 30-F11, lot:9338911)
CD25-PE-Cy7 (BD Bioscience, 552880, PC61, lot:0174949)
CD3-PE/Dazzle 594 (BD Bioscience, 100347, 145-2C11, lot:B281871)
CD11b-e450 (eBioscience, 48-0112-82, M1/70, lot: 2198693)
Gr-1-PE (BioLegend, 108407, RB6-8C5, lot:B275222)
F4/80-APC (BioLegend, 123116, BM8, lot:B298926)
CD206-PE-Cy7 (BioLegend, 141720, C068C2, lot: B353129)
MHCII-FITC (BioLegend, 107605, M5/114.15.2, lot:B323588)

Validation

All the antibodies were validated by manufacturer, and the information about the validation can be found on the manufacturer's website through the links below:
https://www.biolegend.com
https://www.abcam.com
https://www.thermofisher.com/cy/en/home/brands/invitrogen.html
https://www.bdbiosciences.com/en-eu

Eukaryotic cell lines

Policy information about cell lines and Sex and Gender in Research

Cell line source(s)

4T1 (ATCC CRL-2539TM)
E0771 (94A001, CH3 BioSystems)
MCA205 (SCC173, Millipore)

Authentication

No authentication was performed.

Mycoplasma contamination

Cell lines were tested negative for mycoplasma contamination.

Commonly misidentified lines (See ICLAC register)

No commonly misidentified lines were utilized in this study.

Animals and other research organisms

Policy information about studies involving animals; ARRIVE guidelines recommended for reporting animal research, and Sex and Gender in Research

Laboratory animals	C57BL/6OlaHsd, female mice, 6-8 weeks old and BALB/cOlaHsd, female mice, 6-8 weeks old were used in the study. All mice were maintained in specific pathogen-free conditions in the animal facilities of Cyprus Institute of Neurology and Genetics. They were housed in controlled temperature/humidity (22°C/55%) environment on a 12-h light-dark cycle and kept with free access to food and water throughout the whole experiment period.
Wild animals	The study did not involve any wild animals.
Reporting on sex	Experiments were performed with female mice since we used a model for breast cancer.
Field-collected samples	The study did not involve samples collected from the field.
Ethics oversight	All in vivo experiments were conducted in accordance with the animal welfare regulations and guidelines of the Republic of Cyprus and the European Union (European Directive 2010/63/EE and Cyprus Legislation for the protection and welfare of animals, Laws 1994-2013) under a license acquired and approved (No CY/EXP/PR.L2/2018, CY/EXP/PR.L14/2019, CY/EXP/PR.L15/2019, CY/EXP/PR.L03/2020) by the Cyprus Veterinary Services committee, the Cyprus national authority for monitoring animal research for all academic institutions.

Note that full information on the approval of the study protocol must also be provided in the manuscript.

ChIP-seq

Data deposition

☐ Confirm that both raw and final processed data have been deposited in a public database such as GEO.

☐ Confirm that you have deposited or provided access to graph files (e.g. BED files) for the called peaks.

Data access links<br>*May remain private before publication.*	*For "Initial submission" or "Revised version" documents, provide reviewer access links. For your "Final submission" document, provide a link to the deposited data.*
Files in database submission	*Provide a list of all files available in the database submission.*
Genome browser session<br>(e.g. UCSC)	*Provide a link to an anonymized genome browser session for "Initial submission" and "Revised version" documents only, to enable peer review. Write "no longer applicable" for "Final submission" documents.*

Methodology

Replicates	*Describe the experimental replicates, specifying number, type and replicate agreement.*
Sequencing depth	*Describe the sequencing depth for each experiment, providing the total number of reads, uniquely mapped reads, length of reads and whether they were paired- or single-end.*
Antibodies	*Describe the antibodies used for the ChIP-seq experiments; as applicable, provide supplier name, catalog number, clone name, and lot number.*
Peak calling parameters	*Specify the command line program and parameters used for read mapping and peak calling, including the ChIP, control and index files used.*
Data quality	*Describe the methods used to ensure data quality in full detail, including how many peaks are at FDR 5% and above 5-fold enrichment.*
Software	*Describe the software used to collect and analyze the ChIP-seq data. For custom code that has been deposited into a community repository, provide accession details.*

Flow Cytometry

Plots

Confirm that:

☒ The axis labels state the marker and fluorochrome used (e.g. CD4-FITC).

☒ The axis scales are clearly visible. Include numbers along axes only for bottom left plot of group (a 'group' is an analysis of identical markers).

☒ All plots are contour plots with outliers or pseudocolor plots.

☒ A numerical value for number of cells or percentage (with statistics) is provided.

Methodology

Sample preparation	E0771 breast tumors were harvested in 1x PBS, minced to fine fragments and incubated with Accumax (Millipore) for 1 h at RT on an end-over-end shaker. Enzymatic digestion was ceased by the addition of RPMI media containing 10% FBS and 1% antibiotic /antimycotic solution. The resulting tissue homogenates were filtered through 40 µm cell strainers and single cells suspensions were collected and counted.
Instrument	BD FACSAria III
Software	BD FACSuite software
Cell population abundance	Prior to experiment, we performed compensation using compensation beads to set voltages and gating parameters for obtaining accurate fluorescence signal. For the compensation we used negative and single positive samples. We also used FMOs and isotype controls in the staining panel to ensure the purity of the gated populations. At least 200000 events were acquired from each sample. The exact number of cells corresponding to the acquired events cannot be determined because we did not use counting beads in our experiment.
Gating strategy	Singlets were gated on all events (FSC-H vsFSC-A). Live cells (FVD-e780 negative signal) were gated on singlets. Heat-killed cells were used to determine the boundaries of positive FVD staining. CD45 lymphs were gated on live cells. Percentages of intratumoral CD3+ CD4+ (SP, single positive) cell population and percentages of intratumoral CD3+ CD8+ (SP, single positive) cell population gated on CD45+ lymphs in different treatment groups. regulatory T cells (Tregs) defined as Foxp3 +CD127loCD25hi CD4 SP were gated on CD45+ lymphocytes. Isotype controls or FMOs were used to define the positive and negative staining. Percentages of intratumoral MDSCs (Gr-1+ CD11b+) were gated on CD45+ lymphs. Percentages of TAMs (CD45+ CD11b+ GR1- F4/80+) gated on CD45 lymphocytes.Percentage of antitumor M1-like TAMs (CD45+ CD11b+ GR1- F4/80+CD206-MCHII+) gated on total TAM population.

☒ Tick this box to confirm that a figure exemplifying the gating strategy is provided in the Supplementary Information.

Magnetic resonance imaging

Experimental design

Design type	*Indicate task or resting state; event-related or block design.*
Design specifications	*Specify the number of blocks, trials or experimental units per session and/or subject, and specify the length of each trial or block (if trials are blocked) and interval between trials.*
Behavioral performance measures	*State number and/or type of variables recorded (e.g. correct button press, response time) and what statistics were used to establish that the subjects were performing the task as expected (e.g. mean, range, and/or standard deviation across subjects).*

Acquisition

Imaging type(s)	*Specify: functional, structural, diffusion, perfusion.*
Field strength	*Specify in Tesla*
Sequence & imaging parameters	*Specify the pulse sequence type (gradient echo, spin echo, etc.), imaging type (EPI, spiral, etc.), field of view, matrix size, slice thickness, orientation and TE/TR/flip angle.*
Area of acquisition	*State whether a whole brain scan was used OR define the area of acquisition, describing how the region was determined.*

Diffusion MRI ☐ Used ☐ Not used

Preprocessing

Preprocessing software	*Provide detail on software version and revision number and on specific parameters (model/functions, brain extraction, segmentation, smoothing kernel size, etc.).*
Normalization	*If data were normalized/standardized, describe the approach(es): specify linear or non-linear and define image types used for transformation OR indicate that data were not normalized and explain rationale for lack of normalization.*
Normalization template	*Describe the template used for normalization/transformation, specifying subject space or group standardized space (e.g. original Talairach, MNI305, ICBM152) OR indicate that the data were not normalized.*
Noise and artifact removal	*Describe your procedure(s) for artifact and structured noise removal, specifying motion parameters, tissue signals and physiological signals (heart rate, respiration).*
Volume censoring	*Define your software and/or method and criteria for volume censoring, and state the extent of such censoring.*

Statistical modeling & inference

Model type and settings

Specify type (mass univariate, multivariate, RSA, predictive, etc.) and describe essential details of the model at the first and second levels (e.g. fixed, random or mixed effects; drift or auto-correlation).

Effect(s) tested

Define precise effect in terms of the task or stimulus conditions instead of psychological concepts and indicate whether ANOVA or factorial designs were used.

Specify type of analysis: ☐ Whole brain ☐ ROI-based ☐ Both

Statistic type for inference
(See Eklund et al. 2016)

Specify voxel-wise or cluster-wise and report all relevant parameters for cluster-wise methods.

Correction

Describe the type of correction and how it is obtained for multiple comparisons (e.g. FWE, FDR, permutation or Monte Carlo).

Models & analysis

n/a	Involved in the study
☐	☐ Functional and/or effective connectivity
☐	☐ Graph analysis
☐	☐ Multivariate modeling or predictive analysis

Functional and/or effective connectivity

Report the measures of dependence used and the model details (e.g. Pearson correlation, partial correlation, mutual information).

Graph analysis

Report the dependent variable and connectivity measure, specifying weighted graph or binarized graph, subject- or group-level, and the global and/or node summaries used (e.g. clustering coefficient, efficiency, etc.).

Multivariate modeling and predictive analysis

Specify independent variables, features extraction and dimension reduction, model, training and evaluation metrics.
